# Breast cancer patient-derived organoids for the investigation of patient-specific tumour evolution

**DOI:** 10.1186/s12935-024-03375-5

**Published:** 2024-06-27

**Authors:** Serena Mazzucchelli, Lorena Signati, Letizia Messa, Alma Franceschini, Arianna Bonizzi, Lorenzo Castagnoli, Patrizia Gasparini, Clarissa Consolandi, Eleonora Mangano, Paride Pelucchi, Ingrid Cifola, Tania Camboni, Marco Severgnini, Laura Villani, Barbara Tagliaferri, Stephana Carelli, Serenella M. Pupa, Cristina Cereda, Fabio Corsi

**Affiliations:** 1 Dipartimento di Scienze Biomediche e Cliniche, Università di Milano, Via G. B. Grassi 74, 20157 Milan, Italy; 2 Department of Electronics, Information and Bioengineering (DEIB), Politecnico di Milano, 20133 Milan, Italy; 3 Istituti Clinici Scientifici Maugeri IRCCS, 27100 Pavia, Italy; 4 Pediatric Research Center “Romeo and Enrica Invernizzi”, Università di Milano, 20157 Milan, Italy; 5 Center of Functional Genomics and Rare Diseases, Buzzi Children’s Hospital, 20154 Milan, Italy; 6 Microenvironment and Biomarkers of Solid Tumors, Department of Experimental Oncology, Fondazione IRCCS Istituto Nazionale dei Tumori di Milano, 20133 Milan, Italy; 7 Epigenomics and Biomarkers of Solid Tumors, Department of Experimental Oncology, Fondazione IRCCS Istituto Nazionale dei Tumori di Milano, 20133 Milan, Italy; 8 Institute for Biomedical Technologies, National Research Council (ITB-CNR), Via F. lli Cervi 93, 20054 Segrate, Italy

**Keywords:** Breast cancer, Patient-derived organoids, Tumour evolution

## Abstract

**Background:**

A reliable preclinical model of patient-derived organoids (PDOs) was developed in a case study of a 69-year-old woman diagnosed with breast cancer (BC) to investigate the tumour evolution before and after neoadjuvant chemotherapy and surgery. The results were achieved due to the development of PDOs from tissues collected before (O-PRE) and after (O-POST) treatment.

**Methods:**

PDO cultures were characterized by histology, immunohistochemistry (IHC), transmission electron microscopy (TEM), scanning electron microscopy (SEM), confocal microscopy, flow cytometry, real-time PCR, bulk RNA-seq, single-cell RNA sequencing (scRNA-seq) and drug screening.

**Results:**

Both PDO cultures recapitulated the histological and molecular profiles of the original tissues, and they showed typical mammary gland organization, confirming their reliability as a personalized in vitro model. Compared with O-PRE, O-POST had a greater proliferation rate with a significant increase in the Ki67 proliferation index. Moreover O-POST exhibited a more stem-like and aggressive phenotype, with increases in the CD24^low^/CD44^low^ and EPCAM^low^/CD49f^high^ cell populations characterized by increased tumour initiation potential and multipotency and metastatic potential in invasive lobular carcinoma. Analysis of ErbB receptor expression indicated a decrease in HER-2 expression coupled with an increase in EGFR expression in O-POST. In this context, deregulation of the PI3K/Akt signalling pathway was assessed by transcriptomic analysis, confirming the altered transcriptional profile. Finally, transcriptomic single-cell analysis identified 11 cell type clusters, highlighting the selection of the luminal component and the decrease in the number of Epithelial–mesenchymal transition cell types in O-POST.

**Conclusion:**

Neoadjuvant treatment contributed to the enrichment of cell populations with luminal phenotypes that were more resistant to chemotherapy in O-POST. PDOs represent an excellent 3D cell model for assessing disease evolution.

**Supplementary Information:**

The online version contains supplementary material available at 10.1186/s12935-024-03375-5.

## Background

Breast cancer (BC) is the most commonly diagnosed type of tumour and the principal cause of death among women worldwide [1]. This type of cancer is divided into different histological and molecular subtypes [2, 3]. Although the biomolecular classification generally indicates the sensitivity of different tumours to distinct therapies and is a valid tool for defining prognosis, many tumours escape this categorization and have unexpected responses to treatments and a prognosis that is inconsistent with their biomolecular characterization.

Inter- and intra-individual tumour heterogeneity is the major cause of patients’ partial response to anticancer treatments and represents the main obstacle to successful therapy. Indeed, certain treatments are effective for some patients but do not show promising results for others [4]. Although breakthroughs have been made in elucidating the pathobiological complexity of BC, novel molecular and pharmacogenomic markers for predicting drug responses in patients are needed. Therefore, a reliable in vitro preclinical model that closely reflects the original in vivo tumour context may be valuable for assessing the complex biological conditions of BC.

In recent years, three-dimensional (3D) cell cultures have been widely used as preclinical models of numerous diseases, including cancer, to bridge the gap between two-dimensional (2D) cell cultures and animal models [5]. Initially, tumour spheroids were developed and consisted of cell aggregates generated from clones of a single cancer cell, with a specific genetic identity, that grew in suspension. These models had various challenges, mainly due to the lack of tumour cell interactions with the surrounding stromal compartment, which limited their applications as reliable preclinical tumour models. More recently, the tumour patient-derived organoid (PDO) approach, in which the cellular complexity and internal genetic heterogeneity of original cancerous tissue are preserved, has emerged as a very promising tool in translational cancer research and personalized cancer medicine [6]. Organoid cultures consist of clusters of organ-specific cells (stem or progenitor cells) directly derived from fragments of the original tumour. Research has been widely demonstrated that the organoid partially reconstructs the tissue of origin, with a similar structural and functional organization [7]. In BC, PDOs can be successfully derived from all subtypes and show concordance with the corresponding tumour tissue of origin [8]. Indeed, PDO tissue culture has been proposed not only as a model for studying tumour biology but also as a platform for testing different drug therapies for personalized medicine [9, 10]. However, despite the relevant results achieved with colon cancer and other cancer subtypes [11, 12], it is still too early to propose BC-PDOs as a screening platform to predict patient response to therapeutic strategies due to the low success rate and low growth of this type of organoid. In the case of BC, PDOs may be much more useful for studying tumour evolution to resolve the molecular and cellular complexity of the tumour and identify more effective therapies.

In this study, we report the case of a 69-year-old BC patient from whom two PDO cultures were successfully established from specimens collected before (O-PRE) and after (O-POST) neoadjuvant chemotherapy (NACT). Through in-depth characterization of both PDO cultures, we generated a model that was able to recapitulate in vitro patient tumour evolution following NACT, revealing specific cell type gene expression changes and biological and molecular profiles focused on the expression profiles of several biomarkers associated with tumour proliferation and metastasis.

## Methods

### Patient information and sample collection

The patient enrolled at the Breast Unit of the ICS Maugeri IRCCS (Pavia, Italy) according to the protocol of the Bruno Boerci Oncological Biobank (approved by the ethical committee of the ICS Maugeri IRCCS on 27 July 2009) was a 69-year-old woman with a 6 cm ulcerated lesion of left mammary gland. The patient didn’t have a family history for breast or ovarian neoplasia. The biopsy sample was classified as a lobular carcinoma, luminal B molecular subtype (oestrogen 90%, progesterone < 5%, Ki67 60%, HER2 2+ without amplification). At the time of biopsy, after the patient signed the informed consent form, another biopsy specimen was obtained during clip positioning.

### Establishment of PDO culture from biopsy and surgical samples

The biopsy sample obtained from the second biopsy was collected in Ad-DF +  +  + medium (HyClone DMEM-F/12 1:1 supplemented with 10 mM HEPES, 1% penicillin/streptomycin and 1% L-glutamine) and stored at 4 °C until it was processed within 1 h. The specimen was transferred to a Petri dish and finely minced with a scalpel. Then, the sample was collected in a 15 mL tube and digested in 2 mL of Ad-DF +  +  + medium supplemented with 100 µL of 20 mg/mL collagenase (Sigma, C9407) and 2 µL of 10 mM Y27632 (ForLab, M1817) for 3 h at 37 °C. For removal of undigested fragments, the sample was filtered through a 100 µm cell strainer, collected in a 15 mL tube and then centrifuged at 500 × g for 5 min at 8 °C. The supernatant was removed, and the pellet was washed twice. Finally, the pellet was resuspended in 35 µL of cold Cultrex^®^ Ultimatrix Reduced Growth Factor Basement Membrane Matrix (BME) (Bio-Techne, BME0010 [13]), seeded in a prewarmed 24-well plate and transferred to an incubator. After approximately 40 min, the BME-PDOs were solidified, and 250 µL of culture medium (CM; DMEM/F12, 1 × ; L-glutamine, 1%; penicillin/streptomycin, 1%; HEPES, 10 mM; Noggin-conditioned medium, 25 × ; B27 supplement, 1 × ; N-acetyl-cysteine, 1.25 mM; nicotinamide, 0.2 mM; 83–01, 500 nM; Y-27632, 5 µM; R-spondin1-conditioned medium, 10%; Primocin, 50 µg/mL; human EGF, 5 ng/mL; FGF-10, human recombinant, 20 ng/mL; KGF/FGF-7, human recombinant, 5 ng/mL; Heregulin-beta-1, human recombinant, 37.5 ng/mL; and SB, 202190, 500 nM) was added and changed every 2–3 days.

BC surgical tissues were cut into 1–3 mm^3^ pieces, and two random pieces were frozen and stored at −80 °C in the Bruno Boerci Oncological Biobank at the ICS Maugeri IRCCS (Pavia, Italy) for DNA/RNA isolation. Two other random pieces were fixed in formalin and embedded in paraffin for haematoxylin and eosin (H&E) staining and immunohistochemistry (IHC) labelling via routine procedures. Primary O-POST cultures were obtained following the procedure described in a consolidated protocol [13]. Briefly, the collected tissue was removed from the adipose tissue and mechanically and enzymatically digested in 10 mL of Ad-DF +  +  + medium supplemented with 500 µL of 20 mg/mL collagenase and 10 µL of 10 mM Y27632 for 1–2 h at 37 °C. The sample was filtered to remove the undigested tissue, collected in a 15 mL tube and centrifuged. The pellet was washed twice, resuspended in the appropriate amount of BME and seeded in a prewarmed multiwell plate. Once the BME-PDO drops were solidified, the appropriate amount of CM, depending on the multiwell size, was gently added. Every 7–10 days when confluence was achieved, O-PRE and O-POST cultures were collected and passaged.

Each organoid culture was frozen in cell culture freezing medium (Gibco, 12,648–010). Briefly, it is important to dissolve the BME by adding 1 µg/mL dispase (Gibco, 17,105–041) to each well, collect the organoids in a 15 mL tube and centrifuge them at 8 °C and 500 × g for 5 min. After two washes with 10 mL of cold Ad-DF +  +  + , the supernatants were removed, and the pellet was resuspended in freezing medium. First, the vials were transferred to −80 °C, and after approximately 24 h, they were kept in liquid nitrogen for long-term storage.

### Histology and IHC

BME-organoid drops were removed with a sterile cell lifter from the plate and transferred into a mould containing a layer of optimal cutting temperature compound (OCT). Once the drops were included in the OCT, the mould was kept at -80 °C until processing. The OCT-embedded PDOs were sectioned to obtain histological slices of approximately 3 µm thickness. After fixation, the histological slides were stained with H&E and labelled with VENTANA BenchMark ULTRA following automated IHC protocols for oestrogen receptor, progesterone receptor, HER2 (c-ErbB2) and Ki67 [14]. For comparisons between the organoids and the tumour of origin, two random surgical tissue samples were fixed in formalin and embedded in paraffin for H&E and IHC labelling via routine procedures. For HER2 amplification assessment, FISH was performed on OCT-embedded sections using the PathVision HER2 DNA probe kit (Abbott Molecular) according to the manufacturer’s protocol.

### TEM and SEM

For morphological transmission electron microscopy (TEM) analysis, one BME-organoid drop was removed from the plate with a cell lifter and transferred to a 1.5 mL tube containing 1 mL of 2.5% glutaraldehyde in cacodylate buffer for 2 h. The samples were postfixed with 1.5% osmium tetroxide in cacodylate buffer, dehydrated on an ethyl alcohol ascending scale and then incubated in Epon. The procedure is described in a previous protocol [13]. The slices were analysed by a Tecnai Spirit Biotween electron microscope (FEI).

For scanning electron microscopy (SEM) analysis, O-PRE and O-POST were treated with 1 µg/mL dispase (Gibco, 17105–041) to dissolve the BME, fixed in 2.5% glutaraldehyde in cacodylate buffer, washed three times and dehydrated on an ascending ethanol scale from 10 to 100% by centrifuging them at each step. Eventually, they were immersed in hexamethyldisilazane, and two drops of the suspension were placed on a coverslip. Once the hexamethyldisilazane was evaporated, the PDOs were coated with palladium gold and analysed by a Leica S-420 scanning electron microscope.

### Confocal microscopy

For immunofluorescence analysis, 3 × 10^6^ organoids were isolated from the BME by incubating them with 1 µg/mL dispase at 37 °C for 1–2 h. PDOs were collected, washed in phosphate buffer (PBS) three times and fixed with 4% paraformaldehyde (PFA) for 15 min at room temperature (RT). After fixation, the PDOs were washed in PBS three times and then permeabilized using 0.1% Triton X-100 for 10 min at RT. After three washes, the PDO pellets were resuspended in 500 µL of blocking solution containing 2% goat serum/2% bovine serum albumin (BSA) in 1 × PBS for 1 h at RT. PDOs were incubated with primary antibodies in blocking solution for 2 h at RT. We used primary rabbit antibodies against Ki67 (Abcam ab243878, 1:500), EGFR (Genetex GTX35199, 1:200), Vimentin (Genetex GTX100619, 1:500) and HER2 (Cell Signalling Technologies #2165, 1:200). PDOs were washed three times in PBS and incubated with the secondary antibodies anti-rabbit Alexa Fluor 546 (1:300), wheat germ agglutinin (FITC) (1:300), and DAPI (1:10000) in blocking solution overnight at 4 °C. After staining, the PDOs were washed three times in 1 × PBS and seeded on specimen slides in ProLong^™^ Gold mounting medium (Invitrogen, P36935) for acquisition with a Leica SP8 confocal microscope equipped with 405, 488 and 513 nm lasers. Acquisition was performed at 1024 × 1024 dpi resolution.

### Flow cytometry

For CD24, CD44, CD49f and EPCAM staining, organoids bearing approximately 3 × 10^6^ cells were isolated from the BME by incubating them with dispase (1 µg/mL). After collection, PDOs were reduced to single cells through the shearing procedure using TrypLe^™^ Select (1 × ; Gibco, 12563–029). After three washes with Hank’s balanced salt solution (HBSS from HyClone) (SH30268.02), the cells were fixed with 4% PFA for 5–10 min on ice. The fixed cells were washed three times with HBSS supplemented with 2% FBS and aliquoted into four tubes containing approximately 7.5 × 10^5^ cells each. The first tube was labelled with a lineage PE cocktail of antibodies (PE mouse anti-human CD2, Cod. 555327, 1:100; PE mouse anti-human CD3, Cod. 555333, 1:00; PE mouse anti-human CD10, Cod. 555375, 1:100; PE mouse anti-human CD16, Cod.555407, 1:100; PE mouse anti-human CD18, Cod. 555924, 1:100; PE mouse anti-human CD31, Cod. 555446, 1:100; PE mouse anti-human CD64, Cod. 558592, 1:100; PE mouse anti-human CD140b, Cod. 558821, 1:100; BD Biosciences) for 15 min at RT to gate the lineage-positive cells, which were excluded from the analysis. The second tube contained only unstained cells to acquire negative signals. The third tube was labelled for 15 min at RT with a lineage cocktail, FITC mouse anti-human CD24 (Cod. 555427, 1:50, BD Biosciences) and APC mouse anti-human CD44 (Cod. 559942, 1:50, BD Biosciences) to identify the CD24/CD44 cell population, while the fourth tube was labelled with lineage cocktail supplemented with FITC rat anti-human CD49f (Cod. 555735, 1:50, BD Biosciences) and APC mouse anti-human EPCAM (Cod. 347200, 1:100, BD Biosciences) to identify CD49f/EPCAM populations. After staining, the labelled cells were washed three times with HBSS supplemented with 2% FBS and analysed using a CytoFLEX flow cytometer (Beckman Coulter). Acquisition was performed on 20,000 events within the selected region of singlets of viable cells.

For EGFR evaluation, 1.5 × 10^6^ organoids were isolated from the BME, reduced into single cells and fixed as described in the previous paragraph. The fixed cells were washed three times with HBSS supplemented with 2% FBS and transferred to two tubes containing approximately 7.5 × 10^5^ cells each. The tube was labelled with the primary chimeric monoclonal antibody cetuximab (CTX, 1:200) for 15 min at RT. The labelled cells were washed three times with HBSS supplemented with 2% FBS. Both tubes were labelled with the Alexa Fluor 488 (AF488) goat anti-human secondary antibody (Thermo Fisher, 1:300). A tube containing cells labelled with only the secondary antibody was used to determine the region of positivity. After staining, the labelled cells were washed three times with HBSS supplemented with 2% FBS and analysed as described above.

### RNA-Seq and bioinformatic analysis

Three different human samples (one healthy breast tissue, one O-POST organoid, three O-PRE organoids and three FFPE tissues) were subjected to RNA-Seq analysis. PDOs were isolated from BME by adding 1 µg/mL dispase and incubating at 37 °C for 1–2 h. The PDOs were collected in a 15 mL tube and centrifuged at 500 × g and 8 °C for 5 min. The pellet was washed twice, and then, an aliquot (1 mL) of PDO suspension was treated with TrypLe™ Select, reduced to single cells and quantified. A PDO suspension containing approximately 30,000 cells was centrifuged, the supernatant was removed and the pellet was maintained at −80 °C until RNA extraction. The O-PRE pellet (N = 3) was collected for RNA-Seq at two different times, at cell passages 3 and 14, while O-POST (N = 1) has been collected at cell passage 19. RNA was extracted using TRIzol^®^ (Invitrogen) following the manufacturer’s instructions. Nanodrop One C (Thermo Fisher) was used for RNA quantification and quality control. RNA-Seq libraries were prepared with the CORALL Total RNA-Seq Library Prep Kit (Lexogen, Vienna, Austria) using 150 ng total RNAs. The RiboCop rRNA Depletion Kit (Lexogen, Vienna, Austria) was used to remove rRNA. The qualities of the sequencing libraries were assessed with D1000 ScreenTape Assay using the 4200 TapeStation System (Agilent Technologies, Santa Clara, CA, USA) to account for variability in library quality and quantified with a Qubit^™^ dsDNA HS Assay Kit (Invitrogen, Carlsbad, CA, USA). RNA-Seq processing was performed via Illumina NextSeq 500 Sequencing. Raw FastQ files were generated via Illumina bcl2fastq2, version 2.17.1.14 (http://support.illumina.com/downloads/bcl-2fastq-conversion-software-v217.html). The bioinformatic data analysis pipeline processed FASTQ data generated by the Illumina NextSeq sequencer through Unique Molecular unique molecular identifier (UMI) extraction, trimming, alignment and quality control steps. As CORALL libraries contain N12 UMI at the start of Read 1, in the first step, UMI were removed through UMI tools. Then, adapter sequences, poly(A) sequences at the 3′ end of Read 1 and poly(T) sequences the 5′ end of Read 2 were trimmed through Cutadapt software. After UMI extraction and trimming, trimmed reads were aligned through STAR using GENCODE Release 38 (GRCh38.p13) as a reference human genome. Gene and transcript abundance were assessed using FeatureCounts software, with the “stranded forward” option. Differential expression analysis was performed using R package DESeq2. Genes were considered differentially expressed and retained for further analysis with |log2(condition sample/control sample) |≥ 1 and a False Discovery false discovery rate (FDR) ≤ 0.05. The R software was used to generate heatmaps (heatmap.2 function from the R ggplots package) and Volcano plots (EnhancedVolcano function from the R EnhancedVolcano package). Gene set enrichment analysis (GSEA) and overrepresentation analysis (ORA) were conducted using KEGG pathway analyses with the clusterProfiler R package (version 4.2.2).

### Quantitative real-time PCR (qRT‒PCR)

Total RNA was extracted from 1 × 10^6^ of O-PRE and O-POST cells using QIAzol (Qiagen, Hilden, Germany) according to the manufacturer’s instructions. cDNA was reverse-transcribed from 1 µg of total RNA in a 20 µL volume using the High-Capacity RNA-to-cDNA Kit (Thermo Fisher Scientific) and subjected to qRT‒PCR using the Applied Biosystems SYBR Green dye-based PCR assay on the ABI Prism 7900HT sequence detection system (Applied Biosystems, Foster City, CA, USA). HER2, Notch3, Notch4, Vimentin and Ki67 mRNA transcripts were amplified using 200 nM primers. The primer sequences are reported in Table S1. The data were normalized to the GAPDH data using the comparative 2-ΔCt method. Only the HER2 data were normalized to the β-actin data.

For RNA-Seq validation, total RNA was extracted from 1 × 10^6^ cells of O-PRE (cell passage 9) and O-POST (cell passage 12) using TRIzol Reagent^™^ (Invitrogen) in accordance with manufacturer's instructions. Then, 2000 ng of RNA was reverse transcribed using the iScript^™^ Reverse Transcription Supermix for qRT-PCR (Bio-Rad). Real Time PCR was performed with the CFX Connect Real-Time System (Bio-Rad) using Sso SYBR Green Supermix (Bio-Rad). Genes were selected among the most interesting BC-related pathways highlighted by the clusterProfiler analysis. Primers were designed using human gene sequences available from NCBI (www.ncbi.nlm.nih.gov/nucleotide) and selected using NCBI's Primer- BLAST tool at the exon junction level to optimize amplification from RNA templates and avoiding nonspecific amplification products. Primers were designed to have a sequence of approximately 20 bp and generate a PCR product size of maximum 250 bp. The primers used are listed in Table S2. Data were normalized to GAPDH using the comparative 2^−ΔΔCt^ method.

### Drug treatment

For establishment of a cell viability assay, the organoids were sheared 2–3 days before seeding to obtain smaller and more uniform PDOs. The organoids were isolated from the BME by adding 1 µg/mL dispase to each well, and the plate was transferred to an incubator at 37 °C for 1–2 h. Once the BME was dissolved, the organoids were collected in 15 mL tubes and washed twice with Ad-DF +  +  + . An aliquot (1 mL) of PDO suspension was treated with TrypLe™ Select, reduced to single cells and used for the cell count. The PDO suspension was diluted in CM containing 10% BME and seeded at 10,000 cells/well in a 96-well spheroid microplate (Corning, 4520) at a concentration of 200 cells/µL. After 24 h, 4 different concentrations of cetuximab (Erbitux^®^ 5 mg/mL, Merck) and the humanized anti-HER2 monoclonal antibody trastuzumab (Ontruzant^®^ 150 mg, Samsung Bioepis) (both ranging from 0.5 nM to 200 nM) were added to 10 replicates. Untreated cells were used as a negative control. After 3 days of expansion at 37 °C and 5% CO_2_, the Cell Titer Glo 3D Kit (Promega, G9682) was used, according to the manufacturer’s instructions, to measure the ATP content as an indicator of cell viability. Emitted luminescence was read in a microplate reader (PerkinElmer, Victor Nivo Multimode), and the data were analysed using GraphPad Prism 8. This experiment has been performed twice.

### scRNA-seq library preparation and sequencing

After PDO shearing and dissociation at the single-cell level using TrypLe^™^ Select, as previously described, 16,500 cells from each of the two PDOs, O-PRE and O-POST, were processed as recommended in the 10X Genomics^®^ Single Cell protocol (v3.1 chemistry) (10X Genomics, Pleasanton, CA, USA). In detail, for each sample, by individually partitioning thousands of cells into nanoliter-scale gel bead-in-emulsion (GEM) products, cDNAs sharing a common 10X Genomics barcode were generated after 11 PCR cycles. scRNA-seq libraries were prepared starting from the cDNAs by 13 PCR cycles, and 10X Genomics barcodes were used to associate individual reads back to each partition. scRNA-seq libraries were diluted 1:10 and run on TapeStation High Sensitivity D5000 screen tape (Agilent Technologies, Santa Clara, CA, USA) for quality assessment. Finally, the two scRNA-seq libraries were sequenced on an Illumina HiSeq2500 instrument (Illumina, San Diego, CA, USA) in a paired-end run (28 cycles for read1, 91 cycles for read2) to obtain at least 40,000 reads per cell for a total of 400 M reads/sample (considering approximately 10,000 cells recovered for each sample).

### scRNA-seq bioinformatics data analysis

Cell Ranger software (v.7.0.0, 10X Genomics) was used to process the obtained reads files. In detail, FASTQ files were generated from demultiplexed raw base call (.BCL) files through the Cell Ranger *mkfastq* pipeline. The Cell Ranger count pipeline was applied to FASTQ to perform alignment against the GRCh38 human reference genome, quality filtering, barcode processing and single-cell gene UMI counting. The Cell Ranger *aggr* tool was used to aggregate outputs from the two samples from the Cell Ranger count, normalize to the same sequencing depth and then recompute the feature-barcode matrices. Cell filtering, data normalization and unsupervised clustering were carried out using the Seurat R package (v.4.1.1) [15, 16]. Based on the principal component (PC) *vs.* variance plots, the top 10 PCs were retained for further analysis. Genes expressed in fewer than three cells were filtered out, as well as genes with fewer than 200 genes and genes with more than 25% mitochondrial gene counts, since mitochondrial RNAs are markers of cell apoptosis. Single cells were filtered based on the following criteria: nCount_RNA [1000–50000] and nFeature_RNA [200–10000]. The scDblFinder Bioconductor/R package (v.1.8.0, [17]) was used for identifying and removing doublets in the dataset. Finally, the Seurat *LogNormalize* function was used to normalize genes by relying on library sequencing depth followed by log transformation. Furthermore, the data were scaled by regressing on different confounding factors, such as the expression of cell cycle genes, the number of UMIs and the percentage of mitochondrial genes.

### Pseudobulk analysis

A pseudobulk approach starting from scRNA-seq data was used for gene set enrichment analysis (GSEA) to fully account for biological variation due to therapeutic treatments. Read counts from cells with the same PDO (O-PRE or O-POST) combination were summed together to form a pseudobulk sample. GSEA was conducted using KEGG pathway analyses via the clusterProfiler R package (version 4.2.2) with the default parameters [18].

### Cluster identification and refinement

After filtering and regression, the Seurat R package (v.4.1.1) was used to project all the cells onto two dimensions by using the uniform manifold approximation and projection (UMAP) method. For exclusion of batch effects, the two biological samples were processed in parallel, and cells from each sample were transcriptionally profiled. Then, the original Louvain graph-based clustering algorithm was used to cluster cells at a resolution of 0.6. Next, we leveraged a nonlinear dimensional reduction technique to aggregate transcriptionally similar cells, and we removed clusters likely to be of low quality resulting from debris, doublets/multiplets and dead cells. Marker genes for each cell cluster were identified using the Seurat *FindMarkers* function with default parameters.

For determination of the cell cluster identity, known cell type-specific markers were selected from previous scRNA-seq studies described in the literature. A cluster showing distinct high expression levels of known marker genes specific for a particular cell type was considered to carry the identity of that cell type. These markers were sufficient to define all major cell types. All marker gene annotations are provided in Table 2.

### Statistical analysis

Statistical data were evaluated using GraphPad Prism version 8.0a (GraphPad Software Inc., La Jolla, USA). The data are reported as the mean ± standard error of the mean (SEM). The level of statistical significance was set at p = 0.05. For confocal image quantification, flow cytometry and qRT‒PCR data were assessed by two-tailed unpaired Student’s t test. The drug screening results were analysed by two-way ANOVA to determine the effects of both the drug and organoid type. The linear regression slopes of the growth curves were calculated using GraphPad Prism version 8.0a (GraphPad Software, Inc., La Jolla, USA), while the doubling times were determined by nonlinear fitting.

## Results

### Establishment and characterization of PDO cultures

**Fig. 1:**
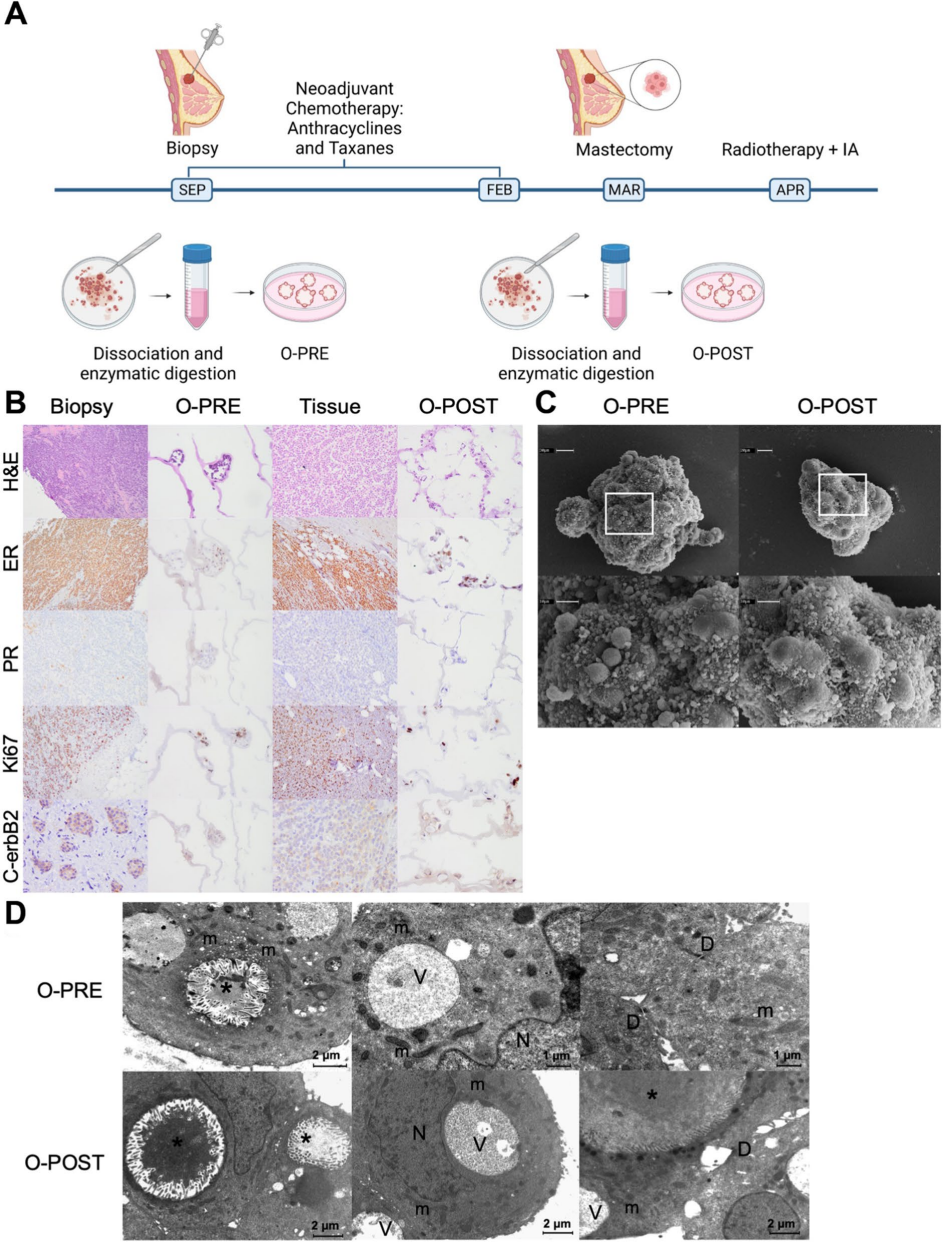
Patient clinical history and characterization of the two tumour tissue samples and matched organoids derived before and after NACT. **A** Timeline of the establishment of the BC PDOs O-PRE and O-POST. **B** Histological and molecular characterization of O-PRE, tumour tissues and O-POST through H&E staining and IHC for oestrogen (ER) and progesterone (PR) receptors, the Ki67 proliferation index and the HER2 (c-erbB2) receptor. O-PRE and O-POST were compared with respect to the histological and molecular features of the tissues of origin. **C** SEM images of O-PRE and O-POST. Both types of organoids showed an almost spheroidal shape. At higher magnification, microvilli and debris are in close proximity to the cell junctions. **D** TEM images of O-PRE and O-POST. They share the same morphological characteristics: numerous intracellular lumens (*) rich in microvilli and containing amorphous material of probable protein origin; elongated mitochondria (m) rich in ridges; and small desmosomes (D) and vacuoles (V). N = nucleus

A 69-year-old woman (Supplementary Material 1: Figure S1, A-E) presented with ulcerated left breast lobular invasive cancer (6 cm) with stage cT2c, 90% oestrogen receptor, < 5% progesterone receptor, 60% Ki67, HER2 2 + (c-erbB2) without gene amplification, cN + (clinical nodes), luminal B-like molecular subtype, and stage III. At this time, a biopsy sample was used to establish O-PRE (Supplementary Material 1: Figure S2). According to tumour histological and molecular characterization, the patient underwent NACT with anthracyclines and taxanes (90 mg/m^2^ epirubicin and 600 mg/m^2^ cyclophosphamide once every 3 weeks (EC) for four cycles followed by 12 cycles of weekly paclitaxel at 80 mg/m^2^). Radiological evaluation revealed > 50% pathological complete response (pCR) in the breast, as observed in Figure S1 (Supplementary Material 1: Figure S1, Panel F). No axillary node response was observed in the preoperative evaluation post-NACT. The patient therefore underwent a left radical mastectomy with complete axillary lymphadenectomy. Pathologic assessment revealed that 8 of 10 lymph nodes were metastatic, with extension to the perinodal adipose tissue. From a surgical sample 3.3 cm in length, a fragment of tissue of approximately 0.5 × 0.5 × 0.5 cm^3^ was used to establish the O-POST PDO (Supplementary Material 1: Figure S2). The biomolecular characteristics of the surgical sample displayed a locally advanced primitive tumour at stage T4b (pTy4b) with involvement of regional lymph nodes (pNy2a) and absence of distant metastasis (pM0); oestrogen receptor 90%, progesterone receptor < 2%, Ki67 20%, and HER2 1+. After the surgery, the patient was treated with locoregional radiotherapy to the left chest wall and ipsilateral clavicular region (dose of 50.4 Gray in 28 fractions with 3D conformal technique). After surgery patient started therapy with aromatase inhibitors (anastrozole). The patient is currently free of disease (Fig. 1A).

To evaluate the effect of NACT on the patient, we generated PDO cultures from PRE- and POST-conditioned samples, as described in the Methods section (O-PRE and O-POST; Supplementary Material 1: Figure S2).

O-PRE and O-POST cultures recapitulated the histological and molecular profiles of the original tissues (Fig. 1B). Indeed, the morphology revealed the features of the original invasive lobular carcinoma (ILC), and the IHC analysis of oestrogen, progesterone, Ki67 and HER2 (c-erbB2) confirmed the molecular characteristics of the biopsy and surgical original tissue. Specifically, O-PRE displayed 40% oestrogen expression, 0% progesterone expression, 60% Ki67 expression and 1% HER2 1+ expression, while O-POST showed 80% oestrogen expression, 0% progesterone expression, 30% Ki67 expression and HER2 2 + expression without gene amplification Fig. 1 and Supplementary Material 1: Figure S3).

We further characterized O-PRE and O-POST morphology by transmission electron microscopy (TEM) and scanning electron microscopy (SEM) to study the ability of PDOs to reproduce the spatial organization of tumour tissue, highlighting how both organoids display typical mammary gland organization. Indeed, the apical secretory portion of the cell faces the interior of the organoid, recapitulating the features of the mammary epithelium [13]. These PDOs are characterized by a 3D well-organized structure with ovoidal cells that are rich in desmosomes. Moreover, they display intercellular lumens and vacuoles that suggest intense secretory activity and are mitochondria-rich in cristae, suggesting that they are involved in functional energy metabolism (Fig. 1C–D). The extensive cell debris extruded at cell junctions on both organoids (Fig. 1D) indicated high secretory activity and cell turnover.

In summary, all these evaluations allowed us to confirm the consistency of O-PRE and O-POST with the tissue of origin.

### O-POST organoids display increased proliferative potential, stemness and aggressiveness

**Fig. 2:**
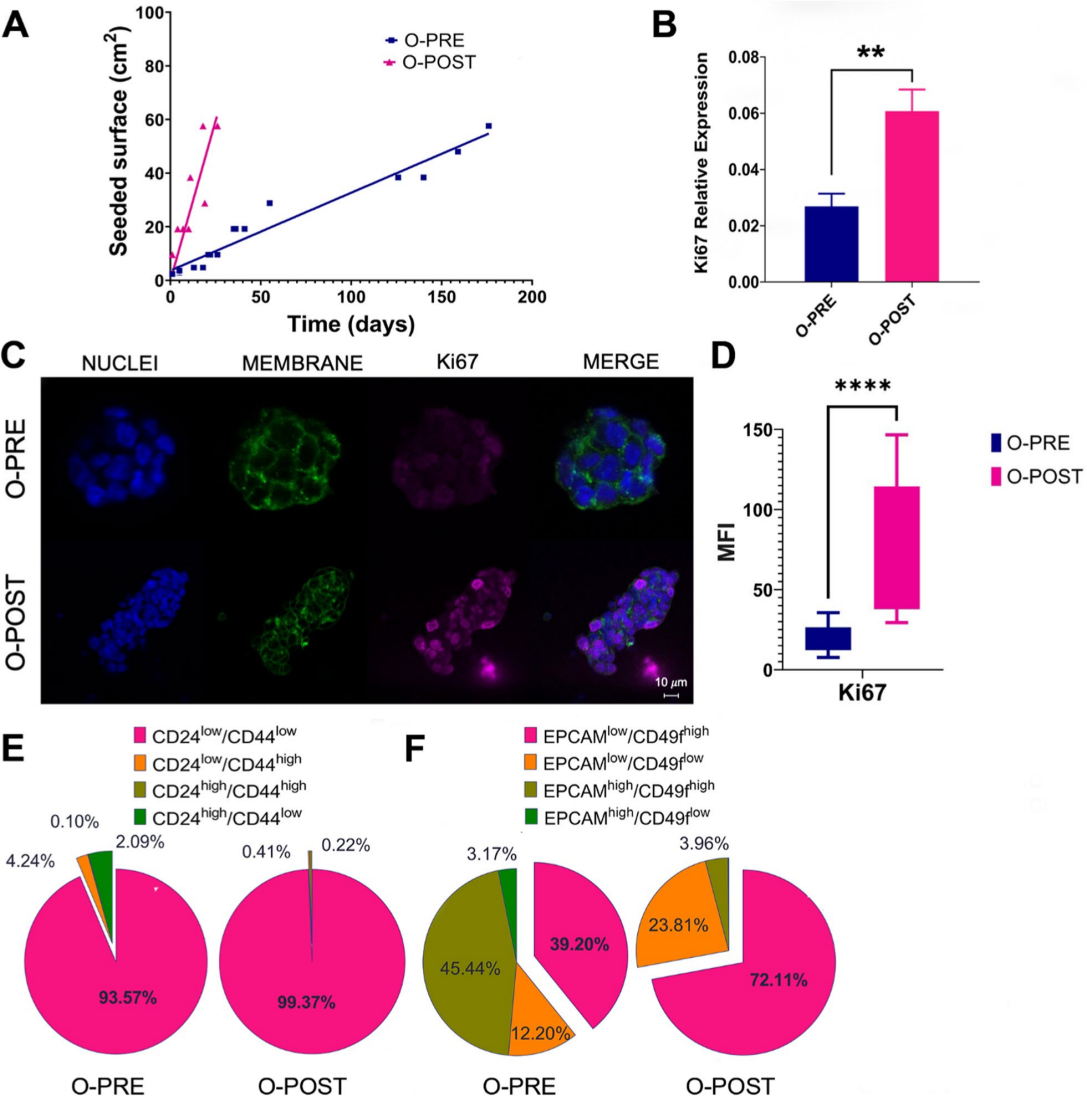
Differences in the proliferation rate and EMT potential between O-PRE and O-POST. **A** Growth curves of O-PRE and O-POST cultures (N = 2–3). (**B**-**D**) Characterization of O-PRE and O-POST by qRT‒PCR (**B**) and confocal microscopy analyses (**C**, **D)** of Ki67 expression. Box plot analysis showing the Ki67 expression levels, evaluated as the relative expression of Ki67 (**B**) and as the mean fluorescence intensity (MFI) (**C**-**D**), obtained from image quantification of O-PRE and O-POST cultures. ****p < 0.0001. Representative images of Ki67 immunofluorescence staining are shown in Panel C. Nuclei (blue, DAPI), membrane (green, WGA FITC) and Ki67 (pink, Anti-Rb AF546) are labelled. Scale bar = 10 µm. **E** O-PRE and O-POST were evaluated by multiparametric flow cytometry for the cell surface markers CD24 and CD44. **F** O-PRE and O-POST were analysed by multiparametric flow cytometry for the expression of EPCAM and CD49f markers

After tissue dissociation and organoid formation, the O-PRE and O-POST cultures exhibited different proliferation rates when expanded in vitro, as shown in Fig. 2A and Supplementary Material 1: Figure S2; moreover, the O-PRE and O-POST cultures exhibited distinct growth patterns even when cultured under the same conditions. In particular, O-POST displayed a 6.5-fold greater slope (0.2851 *vs.* 1.858) and a sixfold lower doubling time (2.388 *vs.* 0.369 days) than O-PRE. Since these PDOs exhibited diverse tumour growth patterns, although cultured under the same conditions, we evaluated their Ki67 proliferation index by qRT‒PCR and confocal microscopy analyses (Fig. 2B‒D). Indeed, O-POST, which exhibited faster growth kinetics, showed a significant increase in Ki67 expression compared with O-PRE (Fig. 2B-D).

The different proliferation rates and, thus, the accelerated in vitro growth capacity shown by O-POST were supported by the greater stemness of the cells that constitute the organoid [19]. Therefore, we evaluated the cell populations linked with the stemness phenotype, i.e.*,* associated with CD24 and CD44 marker expression (Fig. 2E and Supplementary Material 1: Figure S4). Although most studies have reported that the CD24^low^/CD44^high^ population is associated with multipotency, tumour-initiating potential and metastatic properties [20], in the ILC subtype, these features were attributed to the CD24^low^/CD44^low^ cell subset [21], which showed greater representation in O-POST (99.37% ± 0.184) than in O-PRE (93.57% ± 0.368). Moreover, we analysed the expression levels of key markers associated with increased tumour aggressiveness and metastatic potential, such as EPCAM [22, 23]. Specifically, the EPCAM^low^/CD49f^high^ cell population, linked with an elevated probability of metastasis, was enriched among the neoplastic cells that formed O-POSTs (72.11%) (Fig. 2F and Supplementary Material 1: Figure S4).

### Expression of specific surface cancer biomarkers

**Fig. 3:**
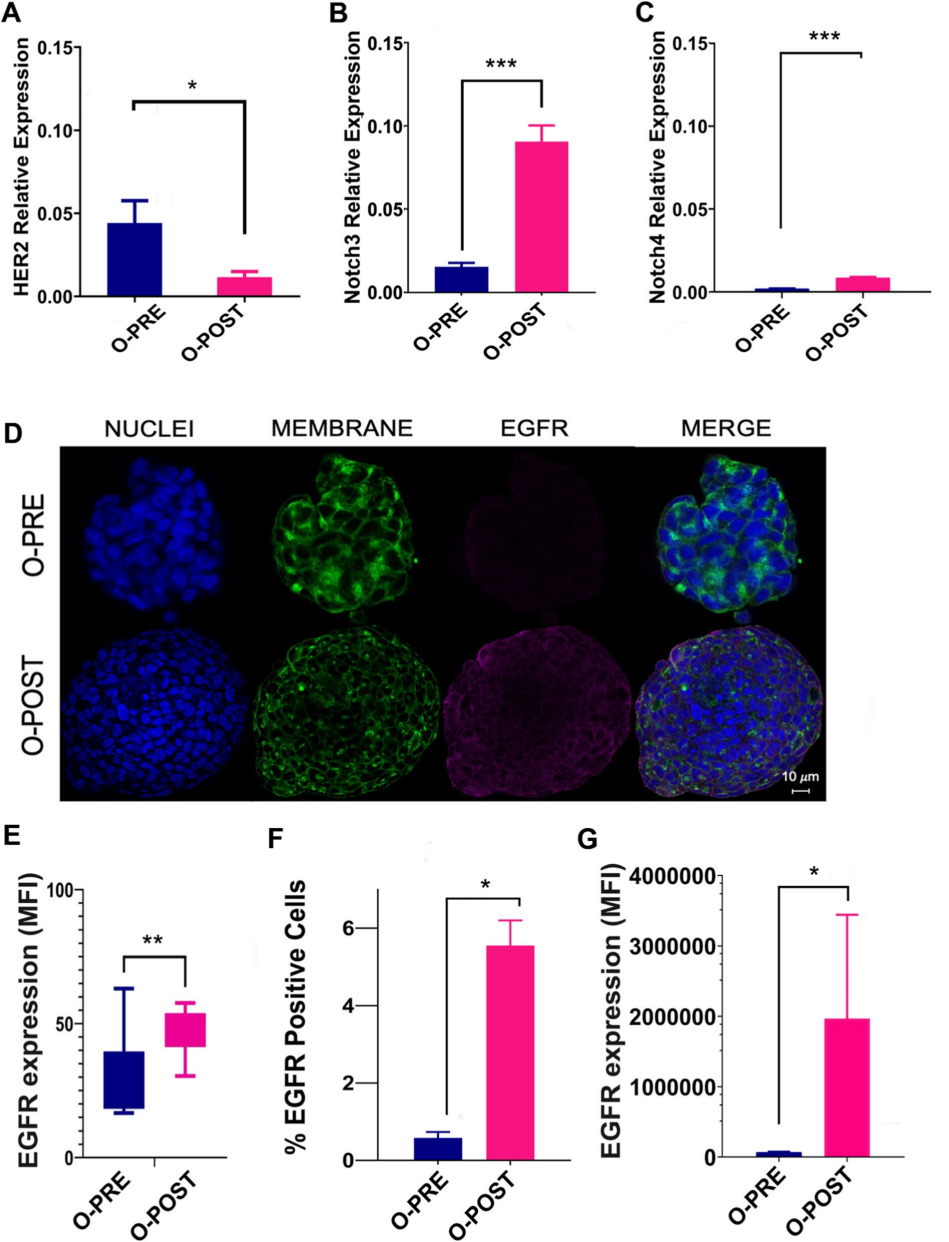
Differential expression of surface cancer biomarkers. **A** qRT‒PCR analysis to evaluate HER2 gene expression was performed with mRNA extracted from O-PRE and O-POST cultures. The data are the means of three independent experiments. The transcript expression levels are presented as the normalized expression. **B**-**C** qRT‒PCR analysis to evaluate Notch3 and Notch4 gene expression was performed with mRNA extracted from O-PRE and O-POST cultures. The data are presented as the means of three independent experiments ± s.e. The transcript expression levels are presented as the normalized expression of β-actin (for HER2) or GAPDH (for Notch3 and Notch4). *p < 0.05; ***p < 0.005. **D** Representative images of EGFR immunofluorescence staining (pink, anti-Rb AF546) in O-PRE and O-POST cultures. Nuclei (blue, DAPI), membrane (green, WGA FITC), and EGFR. Scale bar = 10 µm. **E** Quantification of the EGFR fluorescence signal detected by confocal microscopy in terms of the MFI. **p < 0.01 (p value = 0.004). **F**, **G** EGFR expression in the O-PRE and O-POST groups was evaluated by flow cytometry analysis, using untreated cells to determine the region of positivity and the singlets gate. *p < 0.05

We analysed the main surface BC biomarkers, particularly HER2 and EGFR receptors. As shown by a pathologist, by comparing O-PRE and O-POST, we observed a three-fold reduction in O-POST HER2 protein and mRNA expression (Figs. 1B and 3A), along with an approximately six-fold increase in the relative expression of Notch3 and Notch4 (Fig. 3B, C).

We also observed an increase in EGFR expression in O-POST cells (Fig. 3D–F and Supplementary Material 1: Figure S5), which was confirmed by confocal microscopy and flow cytometry analyses. In this case, a significant difference in EGFR expression between the two organoids was observed. In fact, O-POST resulted in increased EGFR expression on the surface, not only in terms of the mean fluorescence intensity (MFI) but also in terms of the percentage (%) of EGFR-positive cells (Fig. 3E–G and Supplementary Material 1: Figure S5).

### Deregulation of transcriptomic profiles in organoids obtained following neoadjuvant chemotherapy confirms drug responsiveness to specific inhibitors

**Fig. 4:**
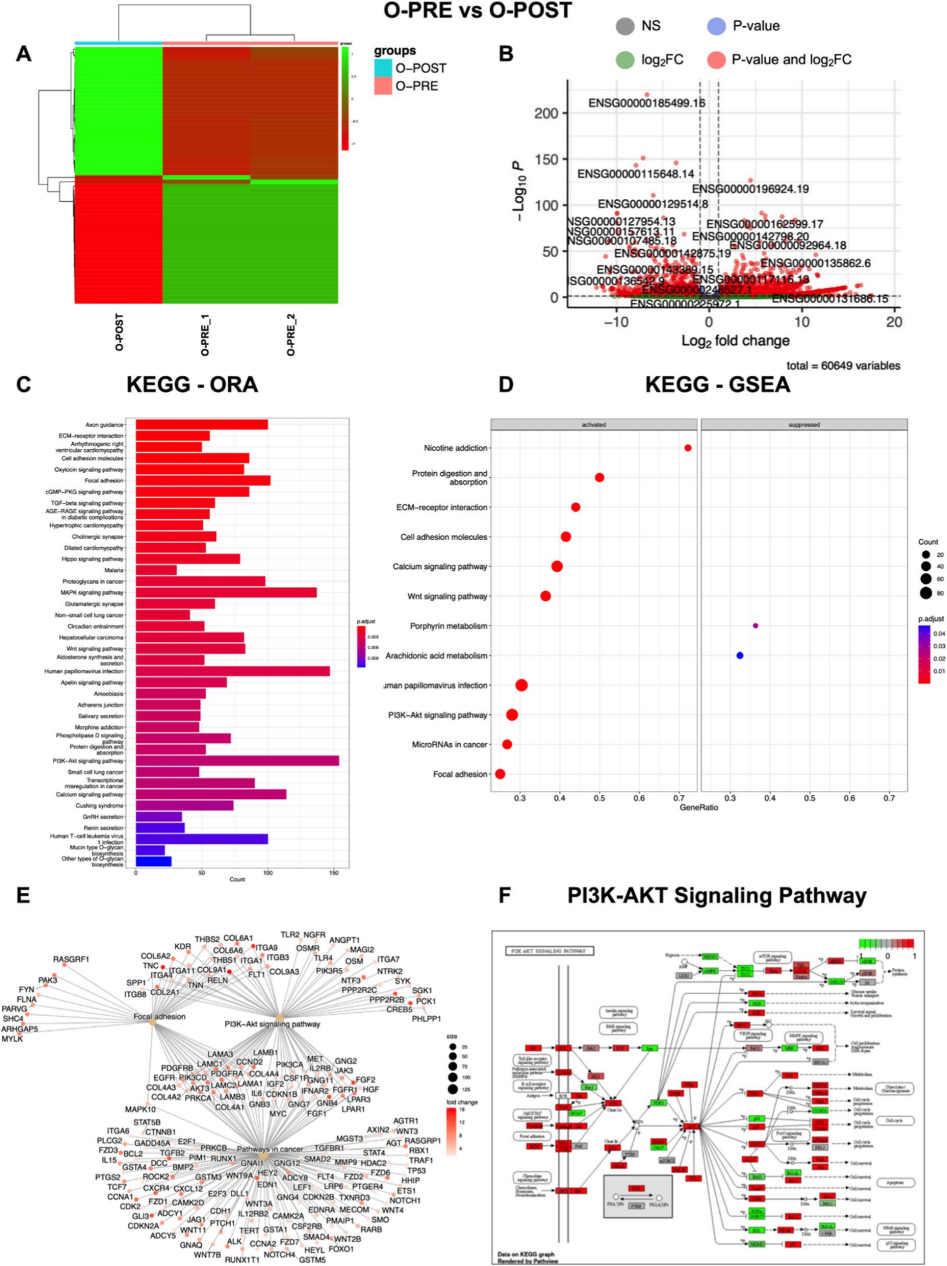
Transcriptome analysis highlights different expression profiles in O-PRE vs. O-POST cultures. We considered as differentially expressed only genes showing |log2(samples/control samples)|≥ 1 and a false discovery rate ≤ 0.05. **A** Heatmap of differentially expressed genes (DE RNAs) in O-PRE vs. O-POST organoid cultures. **B** Volcano plot showing DE RNAs between O-PRE and O-POST. The x-axis shows the log2FC. The p value is shown on a logarithmic scale on the y-axis. Genes that respected the conditions in terms of log2FC and FDR are reported in red, non-DEGs are reported in grey, and genes that respected only one condition are reported either in blue or in green. Considering the 0 on the x-axis, upregulated genes are on the right, while downregulated genes are on the left. **C** Bar plot of the top 40 KEGG pathways in O-PRE vs. O-POST obtained by performing the overrepresentation analysis via clusterProfiler. The y-axis represents the name of the pathway, the x-axis represents the number of DE RNAs in the pathway, and the colour indicates the adjusted p value. **D** Dot plot of KEGG pathways in O-PRE *vs.* O-POST organoid cultures obtained by performing GSEA via clusterProfiler. The figure shows the significantly activated pathways and inhibited pathways. Dot size refers to the number of genes associated with each pathway. The gene ratio is the ratio between the enriched genes and the total genes in the relative pathway database. **E** Cnet plot of specific KEGG pathways in O-PRE *vs.* O-POST organoid cultures obtained by performing GSEA via clusterProfiler. The plot shows the principal node with the name of the specific pathway and the gene of the GSEA core enrichment coloured by log2FC. **F** KEGG pathway analysis of the PI3K-Akt signalling pathway in O-PRE vs. O-POST. The upregulated genes are represented in red, whereas the downregulated genes are represented in green

To further investigate the potential effect of NACT on gene expression in cultured organoids, we focused on O-PRE and O-POST PDO samples. Genes were considered differentially expressed (DE) RNAs and retained for further analysis with |log2(O-PRE/O-POST)|≥ 1 and an FDR ≤ 0.05. Heatmap of the DE RNAs showed different expression profiles (Fig. 4A), as the samples grouped separately. The volcano plot shows the DE RNAs (Fig. 4B), and 3671 DE RNAs were identified; 2260 (61.5%) were found to be upregulated, whereas 1411 (39.5%) were downregulated. Moreover, 90% of the DE RNAs were annotated as coding genes, whereas 10% were noncoding DE RNAs (Table 1). The list of coding DE RNAs, ranked by their fold change (FC), is reported in Supplementary Material as Table S3.

**Table 1:** The number of differentially expressed coding RNAs (mRNAs) and noncoding RNAs (ncRNAs) after transcriptome analysis were divided according to their fold change (FC)

	mRNAs	ncRNAs
Upregulated	2087	173
Downregulated	1263	148
Total	3350	321

To investigate the differences in molecular interactions, we subjected the genes for the O-PRE *vs.* O-POST comparison to overrepresentation analysis with KEGG via clusterProfiler, and the top 40 pathways are shown in Fig. 4C. KEGG analysis demonstrated the deregulation of pathways involved in cytoskeleton regulation, such as “Adherens junction”, “Focal Adhesion” and “Axon guidance”. Moreover, pathways associated with cancer in general (e.g., “Transcriptional misregulation in cancer”, “Small cell lung cancer” and “Proteoglycans in cancer”) and pathways mainly involved in BC, such as the “PI3K-Akt signalling pathway” and “Hippo signalling pathway”, were deregulated (Fig. 4C). Furthermore, the KEGG GSEA performed on clusterProfiler highlighted the activation of “Focal adhesion”, “PI3K-Akt signalling pathway”, “Wnt signalling pathway” and “Pathways in cancer” in patient-derived O-PRE *vs.* O-POST organoid cultures (Fig. 4D, E). The results obtained via RNA-Seq indicated a strong perturbation in the gene expression of central components of the cytoskeleton as well as in pathways involved in BC. Via qRT‒PCR, we analysed and confirmed the expression of 9 genes involved in BC-related pathways, CTNNB, FGF2, GNB4, CDH2, FGFR1, CHRM3, ITGA9, SGK1 and COL4A2 (Supplementary material 1, Supplemental data, Figure S6, Panels A‒I), validating the results of the RNA‒Seq analysis. Moreover, the identification of pathways found to be deregulated in O-PRE *vs.* O-POST by transcriptomic analysis was further corroborated by performing a pseudobulk approach on the scRNA-Seq data, where GSEA confirmed the activation of the “PI3K-Akt signalling pathway”, “Wnt signalling pathway”, “Focal adhesion”, “Pathways in cancer” and “ECM-receptor interaction” pathways (Supplementary Material 1: Figure S7).

**Fig. 5:**
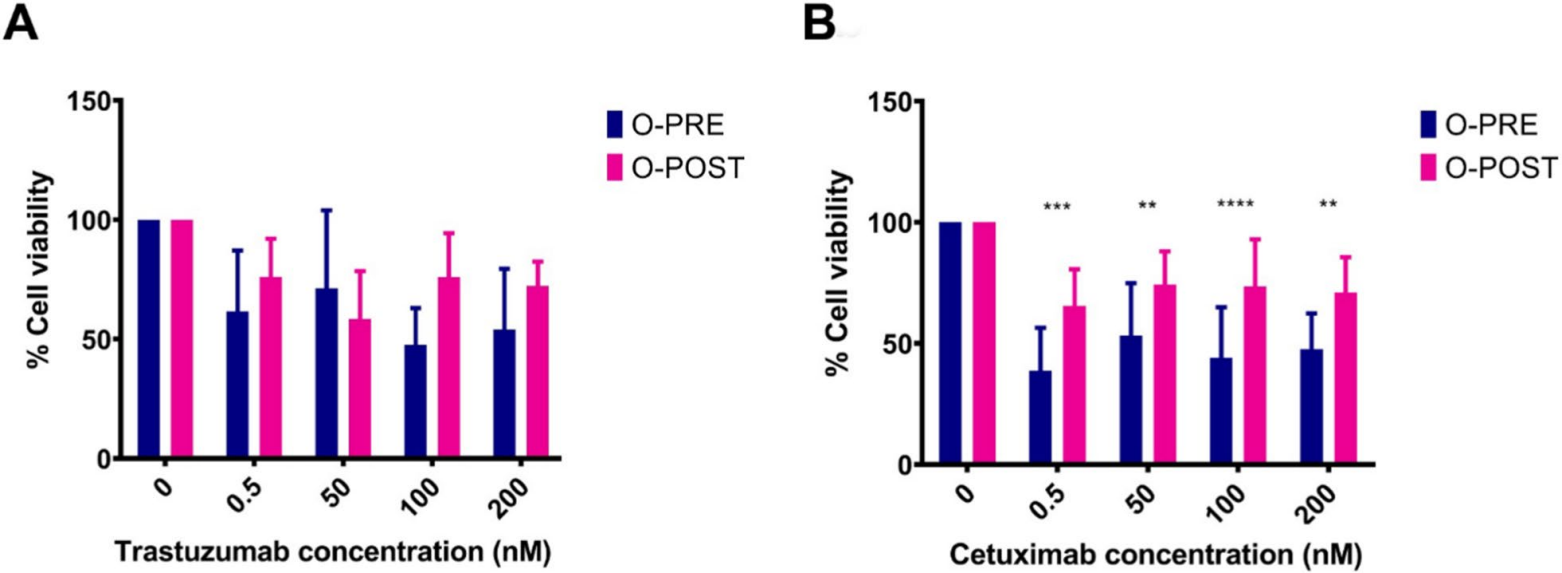
Cell viability assay to assess O-PRE and O-POST drug sensitivity. **A** Drug response of O-PRE and O-POST cultures to treatment with trastuzumab at four different concentrations (0.5 nM, 50 nM, 100 nM, and 200 nM). Ten replicates for each condition were used. **B** Drug response of O-PRE and O-POST to treatment with cetuximab at four different concentrations (0.5 nM, 50 nM, 100 nM, and 200 nM). Ten replicates for each condition were used. The data are reported as the means ± SDs; *p < 0.0332; **p < 0.0021; ***p < 0.0002; ****p < 0.0001

Since these data highlighted deregulation of the PI3K/Akt pathway (Fig. 4F), we performed a cell viability assay to test the effect of two specific inhibitors that target this pathway and are the most commonly employed in the first-line treatment of cancer, despite not being clinically relevant in this specific subset. O-PRE and O-POST were tested with 4 different concentrations (from 0.5 to 200 nM) of trastuzumab and cetuximab, which, respectively target HER2 and EGFR tyrosine kinase receptors upstream of the PI3Ks signalling pathway [24–27]. These drugs mainly displayed cytostatic activity in vitro, preventing the generation of a clear dose‒response curve in vitro since their efficacy is dependent mainly on antibody-dependent cellular cytotoxicity (ADCC). Both organoids were not responsive to treatment directed at the HER2 receptor, and O-POST seemed to be even less sensitive (Fig. 5A). Similarly, we observed a significant increase in EGFR expression in the O-POST group (Fig. 3 C-E). In this case, both O-PRE and O-POST displayed sensitivity to cetuximab, and O-POST, characterized by higher EGFR expression, was significantly less sensitive to cetuximab than was O-PRE, characterized by low EGFR expression, as shown by a cell viability assay (Fig. 5B).

### Single-cell transcriptomics of breast cancer patient-derived organoids

**Fig. 6:**
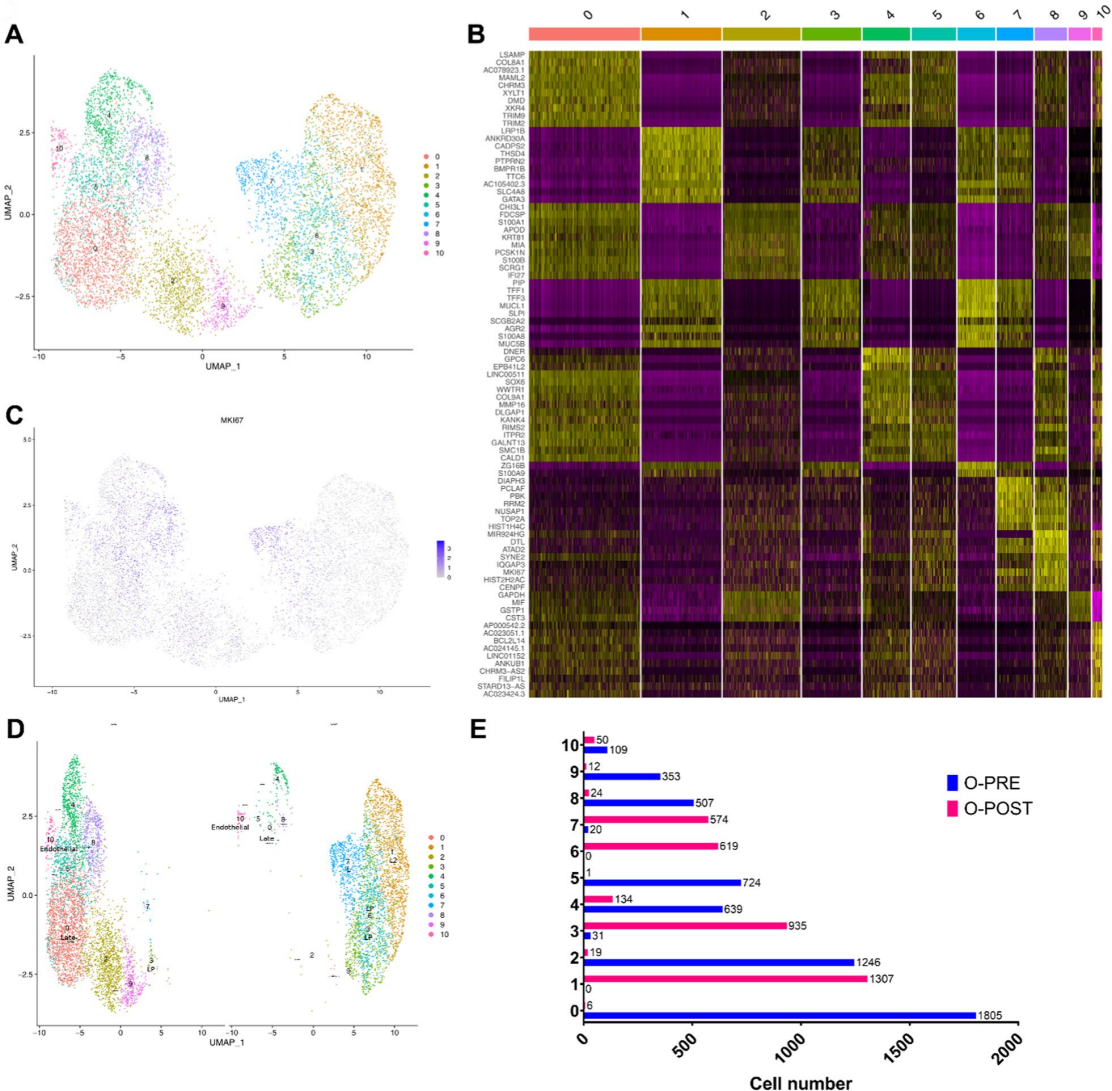
Single-cell RNA-seq analysis of O-PRE and O-POST to characterize cellular populations. **A** UMAP visualization of the 11 identified cell clusters. **B** Heatmap of the top 10 most highly expressed genes in each cluster. **C** UMAP visualization of *MKI67* expression. **D** UMAP representation of principal cell types and related clusters in O-PRE and O-POST organoids. **E** Histogram showing the number of cells in each cluster

For further characterization of the transcriptome profile of PDOs at the single-cell level, a total of 16,500 isolated cells were obtained and counted from each single-cell suspension derived from O-PRE and O-POST. The numbers of recovered cells, reads, genes and transcripts/cells are reported in Table S4. After filtering, the total number of cells obtained from the two samples was 9115, and the cells were separated into 11 cell clusters (Fig. 6A). The genes most highly expressed in each cell cluster compared to all the others were identified using the Seurat *FindMarkers* function (Table S5; Fig. 6B). Cell type identification was carried out by using marker genes selected from the literature and reported in Table 2. Clusters were categorized into seven cell types or subtypes typical of BC. Given the data available in the literature, we identified the two main BC epithelial (*EPCAM*-positive) cell types: luminal epithelial (KRT18-positive) and basal/myoepithelial cells (*KRT14*-positive) (Supplementary Material 1: Fig. S8). Specifically, we identified Clusters 1, 3, 6, and 7 as luminal epithelial cells, as they expressed typical markers, such as the *KRT18* and *KRT8* genes, while Clusters 2, 5, 8 and 9 were identified as basal/myoepithelial cells, marked by the *KRT14, KRT5* and* KRT17* genes. Moreover, among the luminal epithelial cells, we highlighted specific luminal cell subtypes, such as the luminal hormone responsive (L2) subtype (Cluster 1), which expresses the *ESR1, PIP, AGR2*, and* ANKR30A* marker genes, and the luminal progenitor (LP) subtype (Clusters 3 and 6), which is marked by the *CLDN4, S100A8* and *S100A9* genes. Interestingly, Clusters 7 and 8 were highly proliferative with increased expression of the *MKI67* gene, a typical marker of proliferation (Fig. 6C). In addition to luminal and basal BC cell types, we also identified other cell types. Cluster 10 expressed the *LAMA4,* *NRP2, BDP1* and* CLIC4* genes, which are typical of endothelial cells but are negative for *EPCAM*, *KRT14* and *KRT18* (Supplementary Material 1: Fig. S8). Cluster 4 showed the expression of the *TCF4*, *DNER*, and *GPC6* genes, which promote epithelial–mesenchymal transition (EMT) and are associated with an early state of EMT.

**Table 2:** Cell number distribution in the 11 clusters and marker genes specific for the 7 cell types/subtypes

Cell cluster	Number of cells	Marker genes	Cell type/subtype	Reference
1, 3, 6, 7	3486	KRT8, KRT18, KRT19	Luminal epithelial	[34]
1	1307	ESR1, PIP, AGR2, ANKR30A	Luminal hormone responsive subtype (L2)	[35]
3, 6	1585	CLDN4, S100A8, S100A9	Luminal progenitor subtype (LP)	[36]
2, 5, 8, 9	2886	KRT14, KRT5, KRT17	Basal/Myoepithelial	[34]
4	773	TCF4, DNER, GPC6	Early epithelial mesenchymal transition (EMT)	[37–39]
0	1811	VIM, S100A4, CTNNB1	Late epithelial–mesenchymal transition (EMT)/mesenchymal	[40]
10	159	LAMA4, NRP2, BDP1, CLIC4	Endothelial	[41–44]

Cluster 0 was defined as the late-EMT cell type with a mesenchymal phenotype since it expressed typical marker genes such as *VIM, S100A4,* and *CTNN*B1.

By comparing clusters in the O-PRE and O-POST groups, we observed markedly different cellular compositions (Fig. 6D, E)**.** O-PRE strongly enriched the basal/myoepithelial cell type, while in O-POST, there was a predominance of the luminal component, suggesting that NACT selects the luminal component, which is less sensitive to chemotherapy. We highlighted a dramatic decrease in the number of EMT cell types in O-POST organoids (Fig. 6D, E).

## Discussion

In this case study, we successfully established two PDO lines (O-PRE and O-POST) derived from a BC patient. They represent matched organoids derived at different time points during the timeline of the BC patient’s clinical history, i.e., before (O-PRE) and after (O-POST) NACT; thus, they appear to be a reliable tool for studying the biological and genetic cellular evolution of neoplastic disease following therapy. The histology and molecular profiles of O-PRE and O-POST cultures were found to recapitulate the main characteristics of the original tumour tissues, supporting the feasibility of using PDOs as a personalized in vitro 3D tumour model. The molecular comparison between O-PRE and O-POST cultures revealed a markedly aggressive phenotype in O-POST. In fact, the two matched organoids grown under the same conditions, i.e.*,* subjected to the same environmental stimuli, displayed different growth kinetics. This in vitro biological behaviour is directly related to the expression level of the proliferation marker Ki67, which was greater in O-POST than in O-PRE (Fig. 2B–D), although this finding deviated from what was estimated by *the *pathologists on original tissues and on O-PRE and O-POST optical cutting temperature (OCT) compound inclusions (60% *vs.* 30%; Fig. 1). This discrepancy of Ki67 determined in tissues could be most likely due to the effects of the unavoidable selection that occurred when tissue-derived cells were cultured. However, if we consider the scRNA-seq results of the corresponding *MKI67* gene (Fig. 6C), we can see that in O-PRE, the global number of Ki67-overexpressing cells is greater than that in O-POST, despite lower levels of expression, which is consistent with the findings of the pathologist's assessment that revealed a reduction in the percentage of Ki-67-positive cells. Moreover, if we focus only on clusters with high expression of *MKI67* (e.g., Clusters 7 and 8; Fig. 6E), the presence of Cluster 7 in O-POST and of Cluster 8 in O-PRE, both characterized by increased expression of the *MKI67* gene, once again confirms the finding of increased proliferative potential of O-POST.

Moreover, the increased proliferative potential coupled with the expression of other biomarkers associated with stemness features and metastasis highlights the increased neoplastic aggressiveness observed in O-POST. Indeed, we found consistent downregulation of the epithelial marker EPCAM in O-POST (Fig. 2F), indicated by the disappearance of the EPCAM^high^/CD49f^low^ cell population and the relevant reduction in the EPCAM^high^/CD49f^high^ cell population. Notably, the greater percentage of EPCAM^low^/CD49f^high^ cells in O-POST, which is associated with a greater probability of distant metastasis after surgery and shorter disease-free survival (DFS) and overall survival (OS), supports its aggressive phenotype [27, 28]. Furthermore, the expression of the CD24^low^/CD44^low^ population, which reflects the main phenotype of luminal BC and is characterized by tumorigenic and metastatic properties, also supports the inherent multipotency and invasive potential of O-POST [21]. These data are in accordance with the in vivo findings of 8 metastatic lymph nodes out of the 10 examined, despite the scRNA-seq results and clinical assessment of lymph node positivity at diagnosis, suggesting that they probably did not develop during NACT. Indeed, Cluster 4, which included cells that overexpress markers of early EMT, and Cluster 0, which was defined as a late-EMT cell type, drastically decreased and disappeared in O-POST (Fig. 6D, E). Moreover, Cluster 10, which likely contains noncancerous stromal cells with a crucial role in supporting the immunosuppressive microenvironment, was completely removed by NACT.

Given the scRNA-seq data, we could argue that NACT is mainly effective against basal/myoepithelial cells in Clusters 2, 5, 8 and 9, as already demonstrated [29].

This phenomenon results in the selection and enrichment of the luminal component due to NACT, as observed in O-POST. These luminal clusters (1, 3, 6 and 7) were effectively treated by adjuvant therapy, allowing the patient to achieve a disease-free interval (DFI) of 40 months. Indeed, Cluster 1, which contains hormone-responsive luminal cells, is the ideal target of hormone therapy administered to patients in combination with radiotherapy. The enrichment of the luminal progenitor (Clusters 3 and 6) observed in O-POST is in agreement with the increase in multipotency and aggressiveness observed in O-POST.

In this specific context, we can assume that O-POST arises from a tissue in which a more aggressive phenotype has been selected due to the administration of NACT. Therefore, the higher expression of invasiveness markers could explain the lower responsiveness of O-POST to in vitro treatments (Fig. 5), as this is characterized by more aggressive cells.

Since PDOs are also reliable platforms for testing drug efficacy and predicting patient drug response [30, 31], we evaluated the expression of HER-2 and EGFR as cell surface-associated tumour biomarkers to identify the most suitable candidate therapeutic targets in this specific case. More importantly, O-POST was associated with increased expression of EGFR and an increased percentage of EGFR-positive cells, suggesting that NACT upregulates EGFR expression.

Furthermore, based on the identification of different deregulated gene pathways, including the PI3K/Akt pathway, a pro-oncogenic signalling axis acting downstream of HER-2 and EGFR whose activation is heavily involved in the regulation of cell survival, cell cycle progression and cellular growth [32, 33] we tested the ability of trastuzumab and cetuximab to target HER-2 and EGFR, respectively. Although both organoids were similarly and barely susceptible to trastuzumab activity, O-PRE showed significantly greater sensitivity to cetuximab than O-POST. RNA-Seq analysis highlighted global transcriptional deregulation when considering O-PRE with respect to O-POST organoid cultures, suggesting that O-PRE could be a good model for evaluating both disease progression and therapeutic sensitivity. Moreover, GSEA revealed the downregulation of PI3K/Akt pathway components in O-POST, indicating that organoid growth is not dependent on EGFR; rather, proliferation could be driven by another proliferative signalling pathway. This observation could explain why O-POST organoids are less sensitive to pharmacological treatments that are able to block the PI3K/Akt pathway by targeting upstream EGFR. Taken together, these results clearly indicate that in O-POST, a more aggressive phenotype is selected. Despite the major efforts made in understanding the complexity of BC, there still remains a need to study the biology of the tumour and to monitor and predict the patient's response to therapy. The case study reported here supports the reliability of PDO development as a preclinical tool to study in vitro the tumour biology and changes resulting from its evolution. In fact, due to the possibility of obtaining PDOs from the same patient but at different times in her clinical history, we could highlight some differences in proliferative capacity, aggressiveness, and propensity for invasion. These differences could be a consequence of the patient’s NACT, which contributed to the selection of more treatment-resistant cell populations that triggered alternative growth strategies and allowed the development of a more aggressive phenotype. To date, these results further confirm how therapeutic scheduling established by current guidelines is fully suitable to what truly occurs in tumour evolution. Indeed, the adjuvant treatment proposed for that patient after NACT and surgery are fully reliable with the luminal enrichment observed in the molecular landscape depicted by scRNA-seq in O-POST.

### Supplementary Information

Supplementary Materials 1.

Supplementary Materials 2.

Supplementary Materials 3.

## Data Availability

The data are available in a publicly accessible repository (10.13130/RD_UNIMI/XSYQJQ) after publication upon request. The additional experimental data are available in the supplementary material.

## Abbreviations

BC: Breast cancer
BME: Basement Membrane Matrix
CM: Culture medium
cN: Clinical nodes
DE RNAs: Differentially expressed RNAs
EMT: Epithelial–mesenchymal transition
GSEA: Gene set enrichment analysis
IHC: Immunohistochemistry
ILC: Invasive lobular carcinoma
MFI: Mean fluorescence intensity
NACT: Neoadjuvant chemotherapy
OCT: Optimal cutting temperature compound
PFA: Paraformaldehyde
RT: Room temperature
TEM: Transmission electron microscopy
SEM: Scanning electron microscopy
UMI: Unique molecular identifiers
