# Supplementary material for: Breast cancer patient-derived organoids for the investigation of patient-specific tumour evolution
Source: Cancer Cell Int. 2024 Jun 27;24:220. doi: 10.1186/s12935-024-03375-5 (PMC11210105; doi:10.1186/s12935-024-03375-5)
Supplement: Supplementary file 1 — Supplementary Materials 1. [file 12935_2024_3375_MOESM1_ESM.docx]

## Breast cancer patient-derived organoids for the investigation of patient-specific tumour evolution

**Serena Mazzucchelli,^1,*^ Lorena Signati,^1^ Letizia Messa,^2,4,5^ Alma Franceschini,^6^ Arianna Bonizzi,^3^ Lorenzo Castagnoli,^6^ Patrizia Gasparini,^7^ Clarissa Consolandi,^8^ Eleonora Mangano,^8^ Paride Pelucchi,^8^ Ingrid Cifola,^8^ Tania Camboni,^8^ Marco Severgnini,^8^ Laura Villani,^3^ Barbara Tagliaferri,^3^ Stephana Carelli,^4,5^ Serenella M. Pupa,^6^ Cristina Cereda,^5^ Fabio Corsi.^1,3,*^**

1. Dipartimento di Scienze Biomediche e Cliniche, Università di Milano, Milan 20157, Italy.
2. Department of Electronics, Information and Bioengineering (DEIB), Politecnico di Milano, Milan 20133, Italy.
3. Istituti Clinici Scientifici Maugeri IRCCS, Pavia 27100, Italy.
4. Pediatric Research Center "Romeo and Enrica Invernizzi", Università di Milano, Milan 20157, Italy.
5. Center of Functional Genomics and Rare Diseases, Buzzi Children's Hospital, Milan 20154, Italy.
6. Microenvironment and Biomarkers of Solid Tumors, Department of Experimental Oncology, Fondazione IRCCS Istituto Nazionale dei Tumori di Milano, Milan 20133, Italy
7. Epigenomics and Biomarkers of Solid Tumors, Department of Experimental Oncology, Fondazione IRCCS Istituto Nazionale dei Tumori di Milano, Milan 20133, Italy
8. Institute for Biomedical Technologies, National Research Council (ITB-CNR), Via F.lli Cervi 93, 20054 Segrate (MI), Italy

**1. SUPPLEMENTAL TABLES AND FIGURES**

**Table S1**. Primers used for RT-PCR assessment of HER2, Notch, Vimentin and Ki67.

| Primer Name | Primer Sequence |
| --- | --- |
| GAPDH-FW | GCTCACTGGCATGGCCTTC |
| GAPDH-REV | CCTTCTTGATGTCATCATATTTGGC |
| HER2-FW | GTGTGGACCTGGATGACAAGGG |
| HER2-REV | GCTCCACCAGCTCCGTTTCCTG |
| βactin-FW | AGGCATCCTCACCCTGAAG |
| βactin-REV | TCCATGTCGTCCCAGTTGGT |
| Notch3-FW | TGGCGACCTCACTTACGACT |
| Notch3-REV | CACTGGCAGTTATAGGTGTTGAC |
| Notch4-FW | AATGTATGCCCACCAGACCA |
| Notch4-REV | CCTGCCTCATCCTAGCATCT |
| Ki67-FW | TCCTTTGGTGGGCACCTAAGACCTG |
| Ki67-REV | TGATGGTTGAGGTCGTTCCTTGATG |
| Vimentin-FW | TGTCCAAATCGATGTGGATGTTTC |
| Vimentin-REV | TTGTACCATTCTTCTGCCTCCTG |

**Table S2**. Primers used for RT-PCR validation of RNAseq results.

| Primer Name | Primer Sequence |
| --- | --- |
| GAPDH-FW | CTTTTGCGTCGCCAG |
| GAPDH-REV | TTGATGGCAACAATATCCAC |
| CTNNB-FW | AGGTCTGAGGAGCAGCTTCA |
| CTNNB-REV | CCATTGTCCACGCTGGATTT |
| FGF2-FW | CTCATCTCCTGCATGGTGGG |
| FGF2-REV | TGGAGTCAGCAGACACTGTT |
| GNB4-FW | ACACACTCGTGGTCAACTGAA |
| GNB4-REV | AACACGTTTACCCTTCCCAT |
| CDH2-FW | GTGCATGAAGGACAGCCTCT |
| CDH2-REV | TGCAGTTGCTAAACTTCACATTG |
| FGFR1-FW | CTCATCTCCTGCATGGTGGG |
| FGFR1-REV | TGGAGTCAGCAGACACTGTT |
| CHRM3-FW | CTCCTACCTGGAACAGGCCAAC |
| CHRM3-REV | CTTGTGAGTTCCACCACAGGC |
| ITGA9-FW | CACGTCCATCACCGGAATCA |
| ITGA9-REV | TGGGCTGAAAGTGACACTCC |
| SGK1-FW | TTCTCCATCCCGCTCAGTCT |
| SGK1-REV | CACCTTCGTACCCGCTTCT |
| COL4A2-FW | CAGGCCTGTATGGCGAGATT |
| COL4A2-REV | CTCTCAGGTCATGCCCATCC |

**
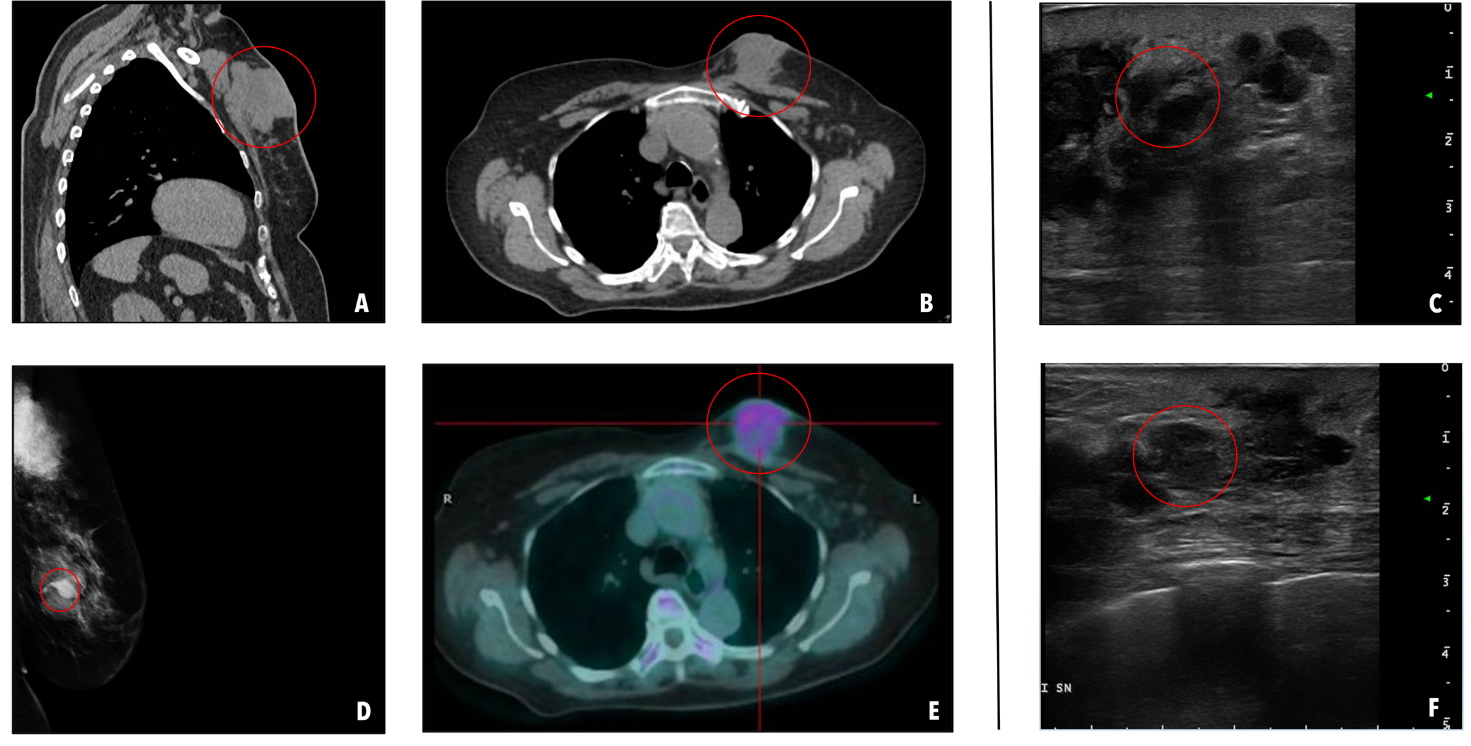
**

**Figure S1.** Radiological imaging before and after neoadiuvant chemotherapy. Chest CT scan (A, B), mammography (D) and Total body PET/CT (E) before NAC. Ultrasound images before (C) and after (F) NAC. Regions of interest are labelled with red circles.


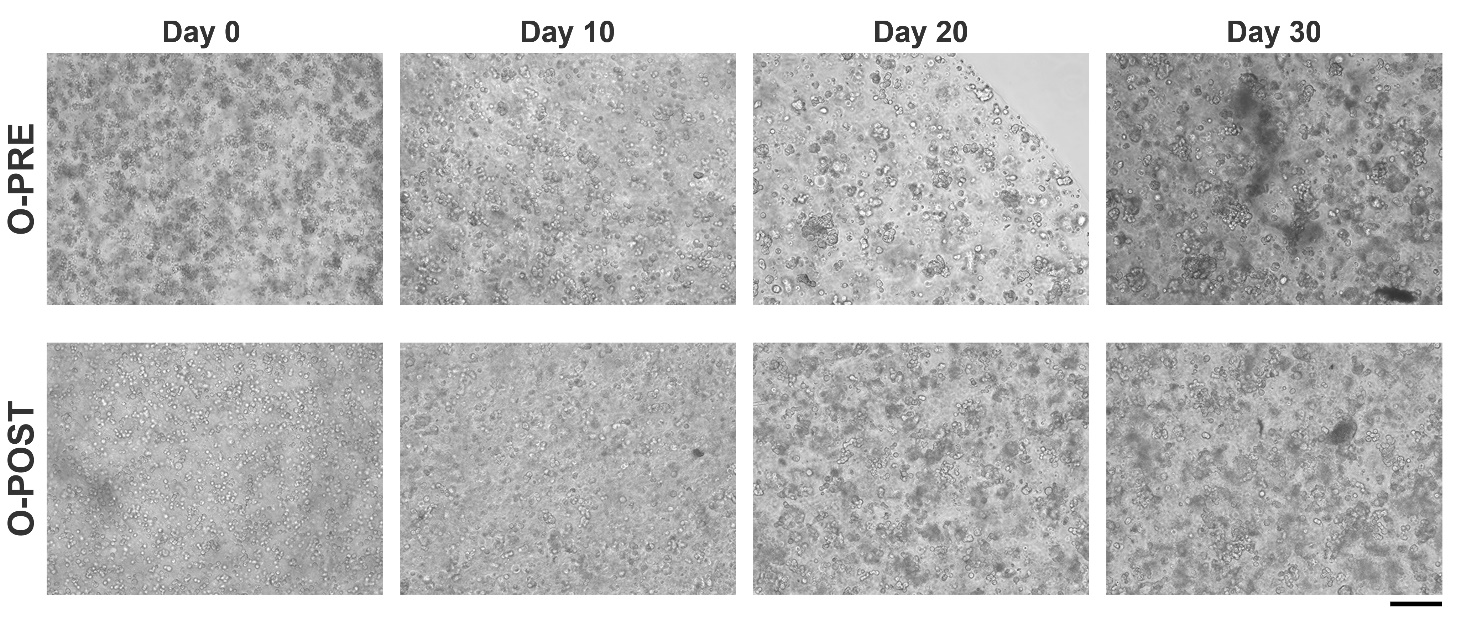


**Figure S2.** Representative images of O-PRE and O-POST at the first seeding achieved after tissue digestion and at the indicated following days.


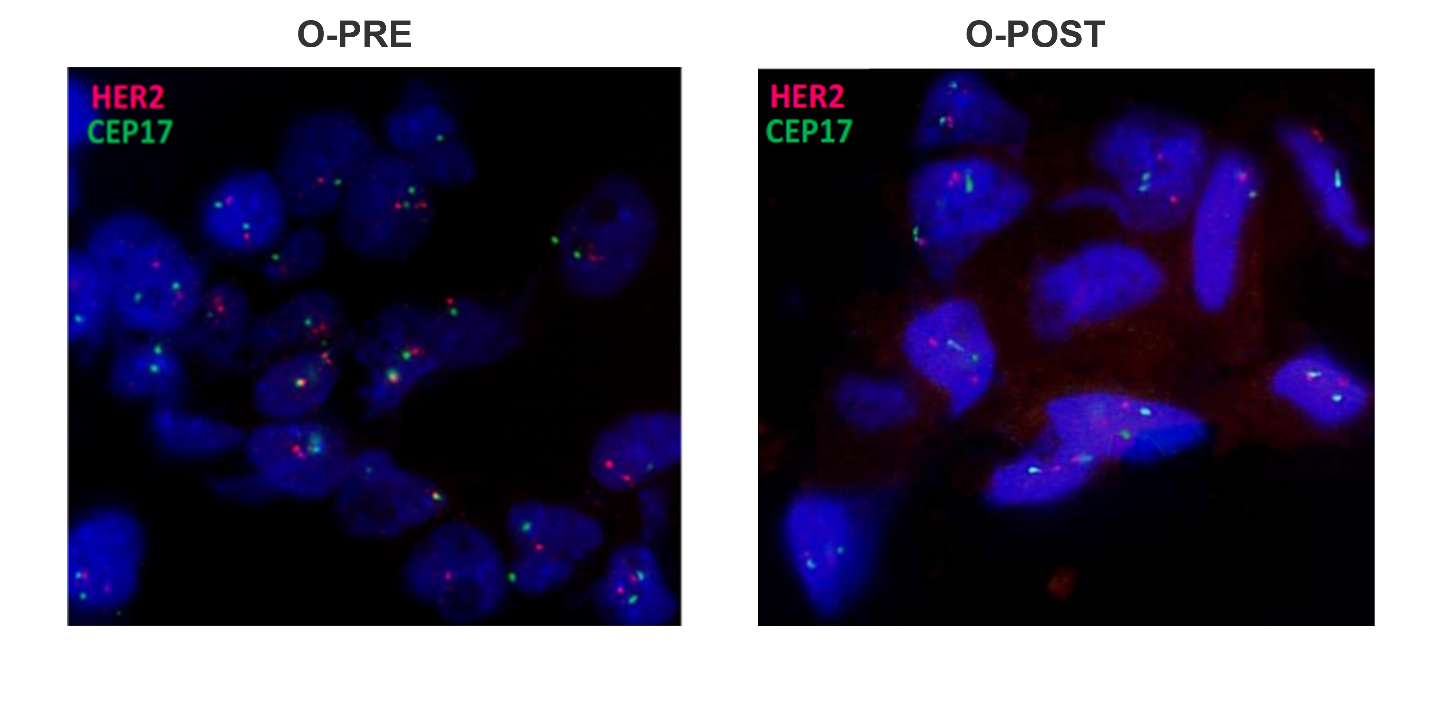


**Figure S3.**  Representative images of O-PRE and O-POST FISH results achieved using the PathVision HER2 DNA probe kit (Abbott molecular). HER2 gene is labelled in red, while the centromeric region of chromosome 17 (CEP17) in green.

**
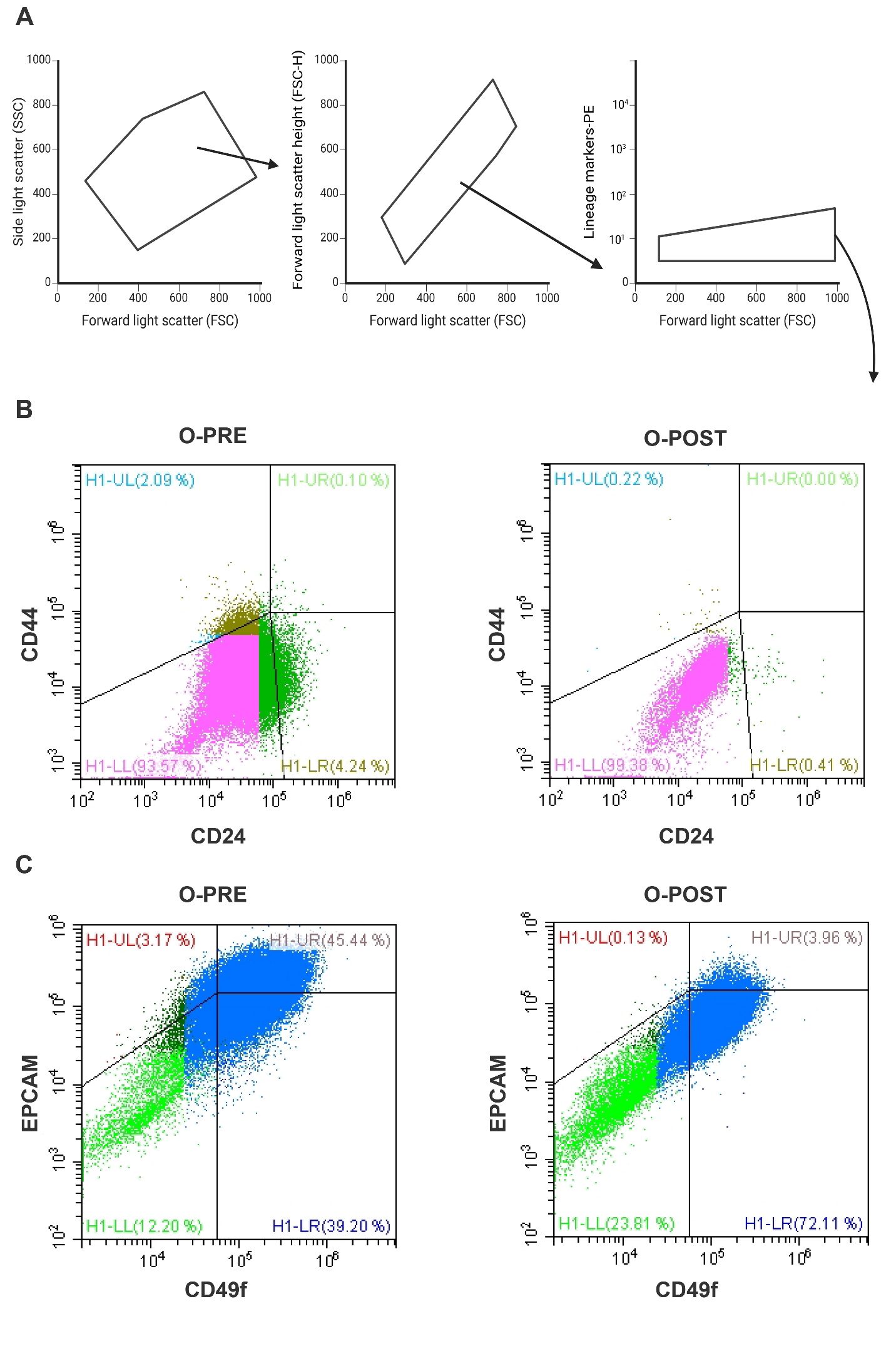
**

**Figure S4.** A) Schematic representation of gating strategy used for CD24-CD44 and CD49f-EPCAM assessment. B) Representative dot-plots of CD24-CD44 results in O-PRE and O-POST. C) Representative dot-plots of CD49f-EPCAM results in O-PRE and O-POST.

**
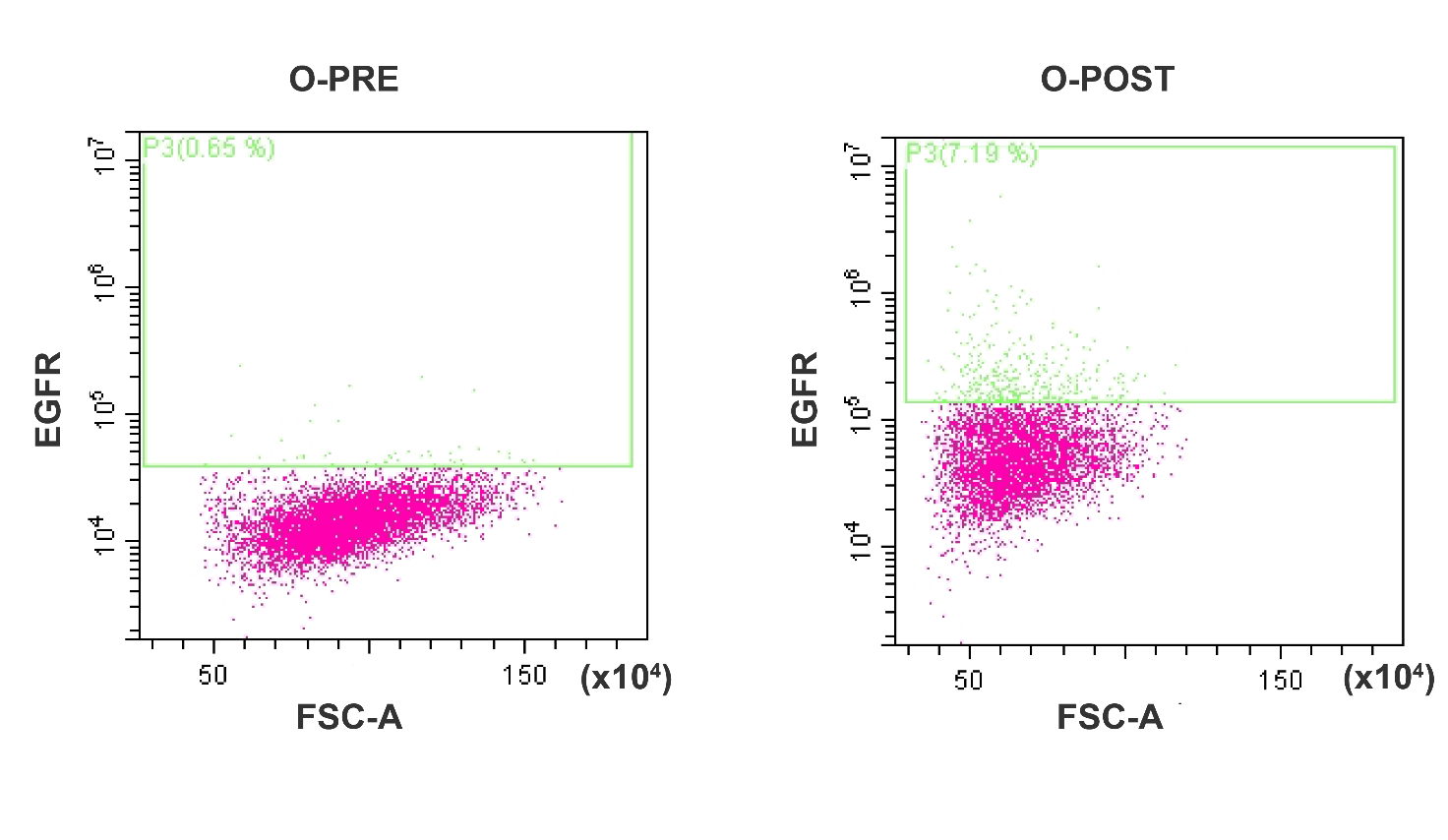
**

**Figure S5.** Representative dot-plots of EGFR expression in O-PRE and O-POST.

**Table S3.** List of coding DE RNAs obtained by RNA seq of O-PRE and O-POST samples, ranked by their Fold Change (FC).

*Please, see the excel file attached as Supplementary Material 2.*

**
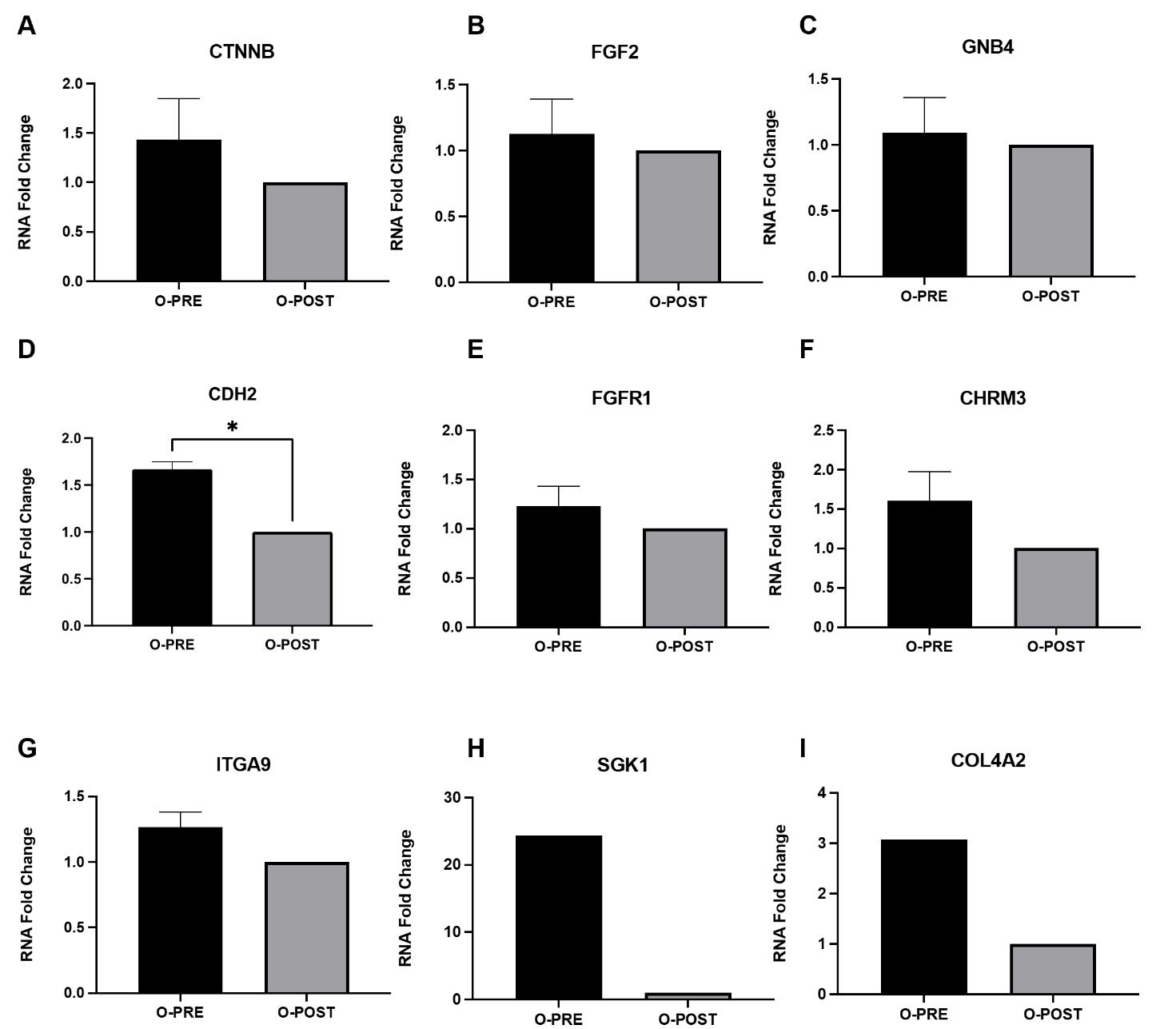
**

**Figure S6.** Expression of CTNNB, FGF2, GNB4, CDH2, FGFR1, CHRM3, ITGA9, SGK1 and COL4A2 genes evaluated by RT-PCR. GAPDH was used as housekeeping gene. Data are expressed as mean of two independent experiments, each performed in duplicate ± SEM, * p < 0.05, vs. O-PRE.


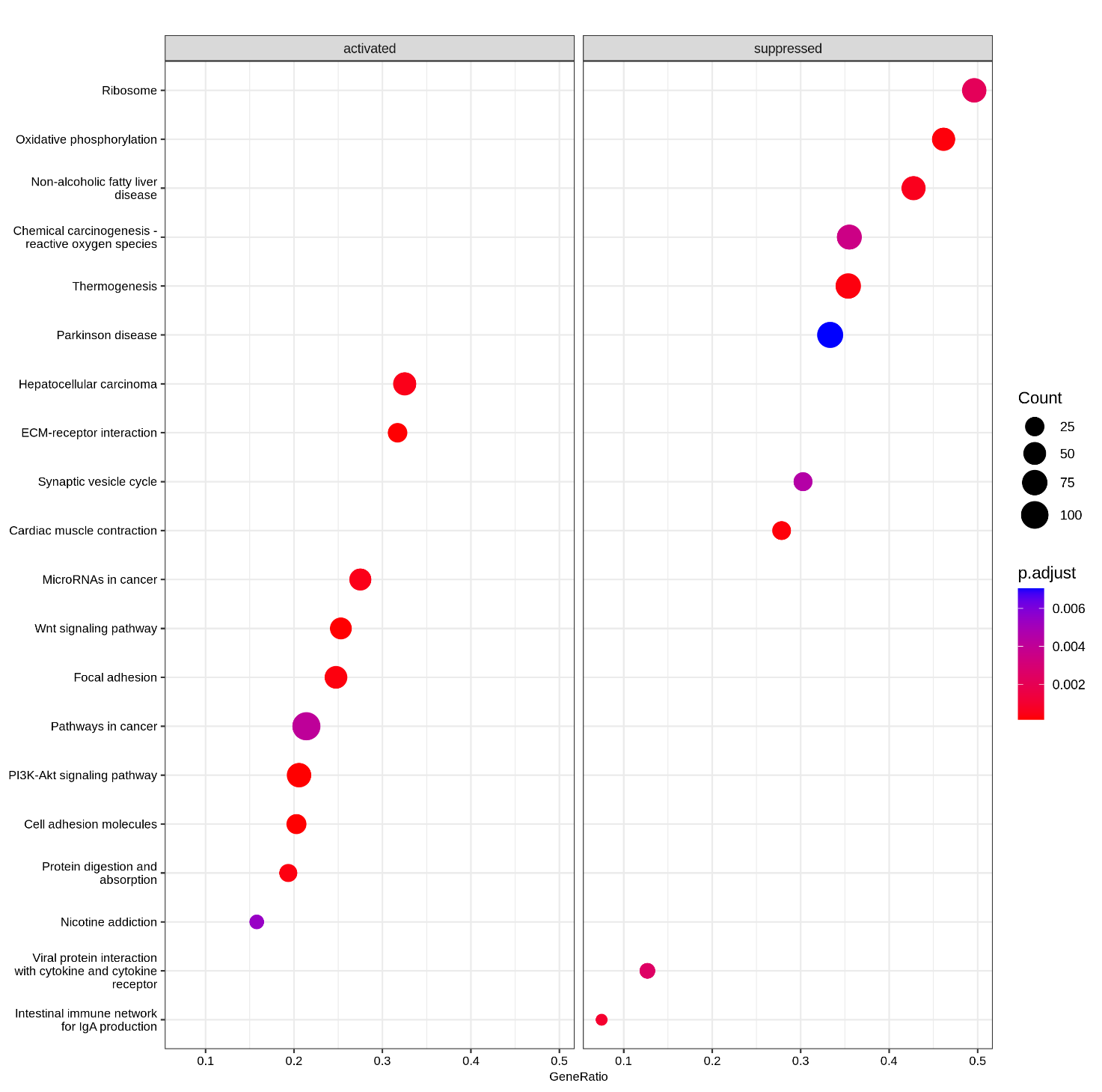


**Figure S7. GSEA dot plots of KEGG pathways in O-PRE vs O-POST organoid cultures**. The figure shows the significant top 10 positively and negatively enriched pathways identified by using pseudo-bulk approach on scRNA-Seq data. Gene count refers to the number of genes associated to each pathway. Gene ratio is the percentage of the enriched genes to the total ones in the relative pathway database.


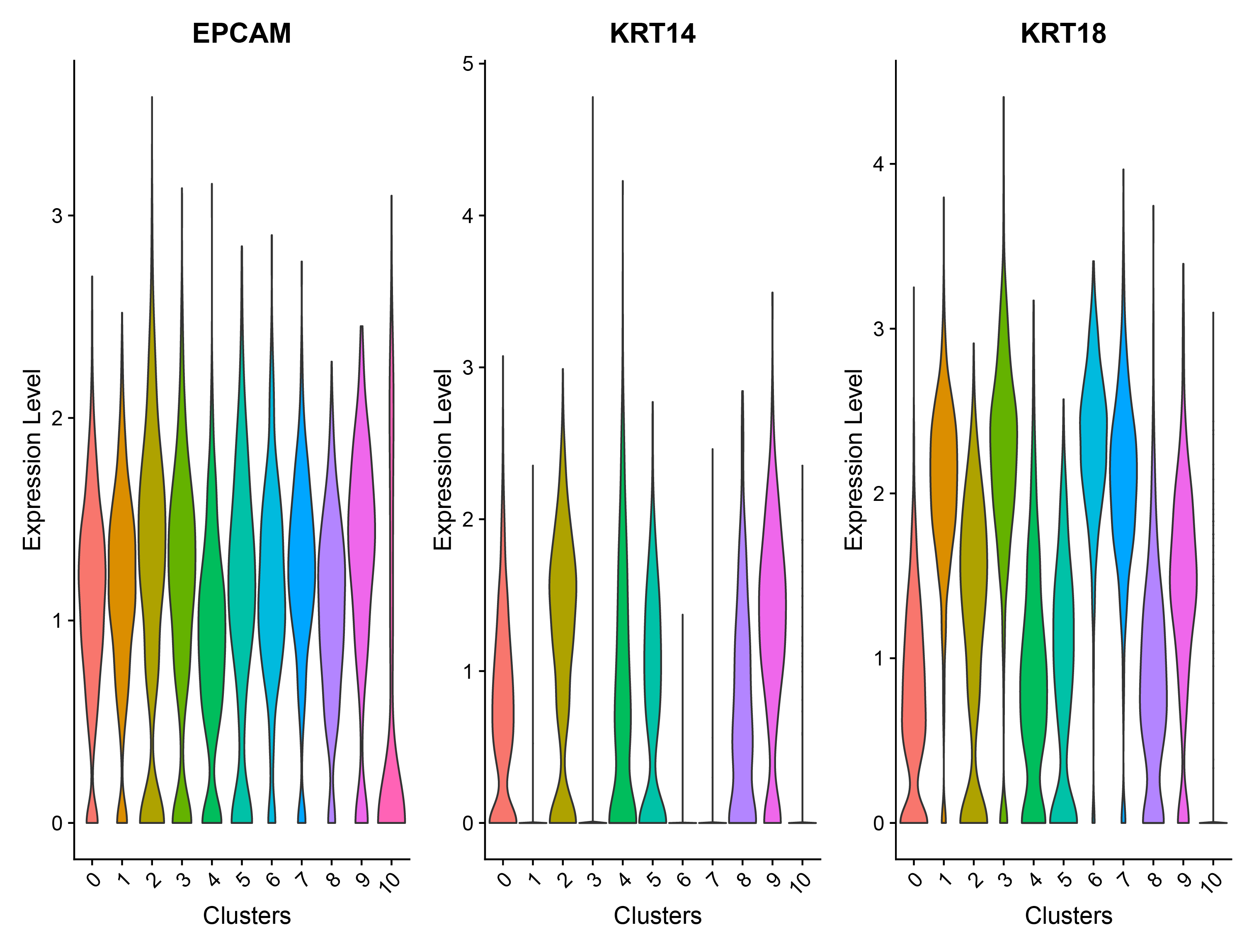


**Figure S8.** Violin plot showing EPCAM, KRT14 and KRT18 expression in each cell cluster.

**Table S4.** Summary of scRNAseq metrics.

|  |  | median per cell | | |
| --- | --- | --- | --- | --- |
| **Samples** | **Cells** | **Reads** | **Genes** | **Transcripts** |
| O-PRE | 7076 | 45269 | 4558 | 13662 |
| O-POST | 5488 | 58809 | 4984 | 18984 |

.

**Table S5.** Genes most expressed in each cell cluster compared to all the others identified by Seurat *FindMarkers* function. *Please, see the excel file attached as Supplementary Material 3.*
